# Characterization of *Toxoplasma gondii* subtelomeric-like regions: identification of a long-range compositional bias that is also associated with gene-poor regions

**DOI:** 10.1186/1471-2164-15-21

**Published:** 2014-01-13

**Authors:** María C Dalmasso, Santiago J Carmona, Sergio O Angel, Fernán Agüero

**Affiliations:** Instituto de Investigaciones Biotecnológicas – Instituto Tecnológico de Chascomús, UNSAM – CONICET, Sede Chascomús, Av. Intendente Marino Km 8, 2 CC 164, B 7130 IWA, Chascomús, Argentina; Instituto de Investigaciones Biotecnológicas – Instituto Tecnológico de Chascomús, UNSAM – CONICET, Sede San Martín, 25 de Mayo y Francia, B 1650 HMP, San Martín, Argentina

**Keywords:** Toxoplasma gondii, Telomeric Associated Sites (TAS), Subtelomeric heterochromatin, Trinucleotide compositional bias

## Abstract

**Background:**

Chromosome ends are composed of telomeric repeats and subtelomeric regions, which are patchworks of genes interspersed with repeated elements. Although chromosome ends display similar arrangements in different species, their sequences are highly divergent. In addition, these regions display a particular nucleosomal composition and bind specific factors, therefore producing a special kind of heterochromatin. Using data from currently available draft genomes we have characterized these putative Telomeric Associated Sequences in *Toxoplasma gondii*.

**Results:**

An all-vs-all pairwise comparison of *T. gondii* assembled chromosomes revealed the presence of conserved regions of ∼ 30 Kb located near the ends of 9 of the 14 chromosomes of the genome of the ME49 strain. Sequence similarity among these regions is ∼ 70%, and they are also highly conserved in the GT1 and VEG strains. However, they are unique to *Toxoplasma* with no detectable similarity in other Apicomplexan parasites. The internal structure of these sequences consists of 3 repetitive regions separated by high-complexity sequences without annotated genes, except for a gene from the *Toxoplasma* Specific Family. ChIP-qPCR experiments showed that nucleosomes associated to these sequences are enriched in histone H4 monomethylated at K20 (H4K20me1), and the histone variant H2A.X, suggesting that they are silenced sequences (heterochromatin). A detailed characterization of the base composition of these sequences, led us to identify a strong long-range compositional bias, which was similar to that observed in other genomic silenced fragments such as those containing centromeric sequences, and was negatively correlated to gene density.

**Conclusions:**

We identified and characterized a region present in most *Toxoplasma* assembled chromosomes. Based on their location, sequence features, and nucleosomal markers we propose that these might be part of subtelomeric regions of *T. gondii*. The identified regions display a unique trinucleotide compositional bias, which is shared (despite the lack of any detectable sequence similarity) with other silenced sequences, such as those making up the chromosome centromeres. We also identified other genomic regions with this compositional bias (but no detectable sequence similarity) that might be functionally similar.

**Electronic supplementary material:**

The online version of this article (doi:10.1186/1471-2164-15-21) contains supplementary material, which is available to authorized users.

## Background

*Toxoplasma gondii* is a widespread obligate intracellular protozoan parasite, member of the phylum Apicomplexa. *T. gondii* has been recognized as an important pathogen for humans, particularly during pregnancy and for immunocompromised patients [1]. Toxoplasmosis has been also documented as an economically important disease that has considerable impact on the livestock industry [1, 2]. It was for these reasons that *T. gondii* was one of the first protozoan parasites chosen for a genome-sequencing project. And more recently, the genomes of other strains of *T. gondii* were sequenced [3].

*T. gondii* presents a haploid genome of ∼63 Mb, which is organized in 14 chromosomes that are well conserved in length and number among different strains [4, 5]. The genomic DNA sequence, and the way it is organized in the nucleus are fundamental for the correct regulation of cell processes. The level of chromatin condensation, its nucleosome composition and positioning, together with their binding to non-histone nuclear proteins generates the different states of chromatin [6]. Euchromatin is a gene-rich decondensed chromatin, where transcription is facilitated, whereas heterochromatin is a gene-poor condensed chromatin, refractory to transcription. A general and very simplified rule is that euchromatin is active chromatin where nucleosomes are enriched in histones H3 and H4 acetylated (H3ac/H4ac), and H3 tri-methylated at lysine 4 (H3K4me3); whereas constitutive heterochromatin nucleosomes are enriched in histone H3 di- and tri-methylated at lysine 9 (H3K9me2/3), which binds Heterochromatin Protein 1 (HP1) forming a compact chromatin. These epigenetic marks are conserved from yeast to humans, and *T. gondii* is not an exception [7–10]. Also, histones H2A and H2B, which are less conserved than H3 and H4, have several variants that contribute differently to the chromatin state [11]. We have previously characterized histones H2A and H2B in *T. gondii*, and found the presence of a histone H2B variant (H2Bv) which is only present in protozoans [12], recently renamed as H2B.Z [13], and two H2A variants: H2A.X and H2A.Z [14]. We have also observed that H2A.Z and H2B.Z are enriched in active promoters, whereas H2A.X is associated to silenced promoters and heterochromatin [14]. Thus, these modified histones and histone variants can be used in *Toxoplasma* as epigenetic markers of euchromatin and heterochromatin.

In constitutive heterochromatin, two specialized domains can be readily identified, the centromere and the telomeres. The centromere is a genetic locus, where the spindle fibers attach to the chromosomes forming the kinetochore, and is required for proper chromosome segregation (reviewed in [15]). Centromeric DNA sequences are not conserved among species, and they differ even between chromosomes in the same organism. However, the centromeric chromatin in all organisms studied to date, contains the histone H3 variant CENP-A [15, 16]. The telomeres, on the other hand, are located at the chromosome ends and contain the telomeric repeats and the subtelomeric region, sometimes denominated Telomeric Associated Sequence (or TAS). Basically, telomeres are composed of a tract of simple repeats (like TTAGGG in humans and trypanosomatids), followed by the subtelomere that comprises repetitive elements and, in some cases, subtelomeric genes [17, 18]. In general, these subtelomeric genes are associated with different stress responses, whose expression is regulated by the Telomeric Position Effect [17]. Telomeres and subtelomeres replicate late in the S-phase of cell cycle [19, 20]. The repetitive elements seem to be involved in blocking replication initiation in the subtelomeric regions [20–22], and favoring their nuclear-periphery localization [20, 23]. Although the features of chromosome ends are highly conserved, the DNA sequences are specific to each species [17]. TASs, as well as centromeres, display a specialized type of chromatin, with a particular nucleosome composition and a set of associated proteins responsible of its highly condensed state [18, 24]. In *Toxoplasma*, only a few heterochromatin-associated proteins have been described to date, including the histone markers mentioned above [8, 14, 25], and the recently described TgChromo1 with a peri-centromeric localization [10]. Concerning constitutive heterochromatin, only centromeres were identified. Centromeric sequences were determined as genomic regions enriched in cenH3 (*Toxoplasma* CENP-A homologous) [26]. Thus, *T. gondii* heterochromatin characteristics, composition, regulation, and distribution across the genome are still to be discovered.

In many protozoan pathogens, such as *Trypanosoma brucei*, *Leishmania major*, *Plasmodium falciparum* and some yeast, the subtelomeric genes are contingency genes [24]. These are sets of genes responsible for pathogen diversity and for the clonal phenotype switches often associated with the parasite’s escape from the host immune system. In the Apicomplexan parasite *P. falciparum*, chromosome ends consist of the telomeric repeat (T(G/A)AAGGG)n followed by a telomere-associated repetitive elements (TARE1-6) organized in six blocks flanked with the members of the *var, stevor* and *rif* multi-gene families responsible for the antigenic variation and cytoadhesion [27–29]. TASs in this parasite also present a specialized chromatin, different from the rest of the genome, rich in nucleosomes containing H3K9me3 and the histone deacetylase PfSir2 [30, 31].

Despite the importance and growing evidence of the effect of telomeres and subtelomeres on the expression of surrounding genes and DNA replication timing modulation, in *Toxoplasma* telomeres have not yet been analyzed; probably because clustered contingency genes at chromosome ends have not been described in this apicomplexan parasite, and/or because the chromosome ends are not completely assembled in the current available genomes. In this study we describe putative subtelomeric sequences in *T. gondii* assembled chromosomes, their nucleosome and nucleotide composition.

## Results

### Several *Toxoplasma*chromosome ends display significant sequence similarity

**Figure 1:**
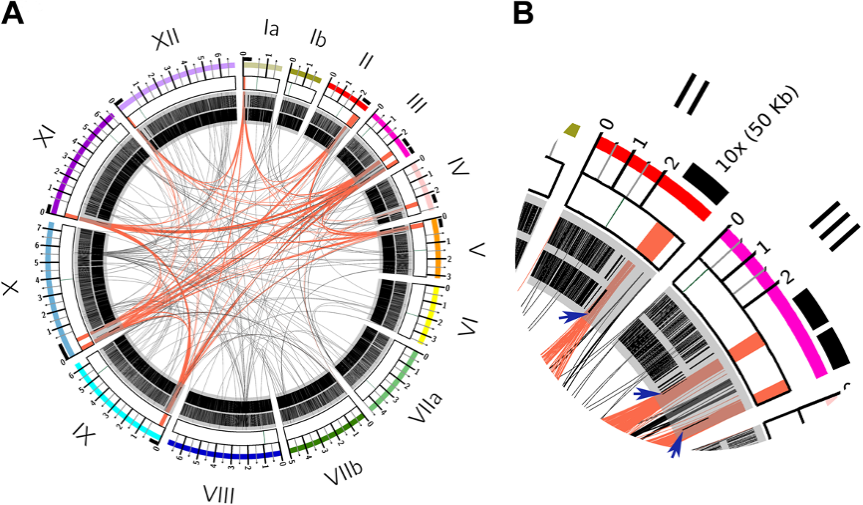
**Telomeric-associated sequences are shared between** ***Toxoplasma*** **chromosome ends.** **A)** Circular ideogram for genome visualization using circos [62]. Chromosomes (I-XII) were laid out in order and labeled with different colors. Centromeres (in green), and TgTAS-like regions (in red) are highlighted in the central white boxes. 50 Kb regions containing the TgTAS-like sequences were zoomed (x10) (see the ruler above chromosomes). Sequence similarity is depicted as ribbons connecting matching regions (red ribbons for TAS-like regions, black ribbons for all other regions). The two inner grey circles show the location of annotated genes in black. **B)** a section of the visualization showing a magnification of chromosomes II and III. Blue arrows indicate the presence of only one gene at the end of each TgTAS-like block.

Subtelomeric sequences show extensive similarity across multiple chromosomes [32, 33]. This has also been observed in other Apicomplexa parasites such as *Plasmodium*[28], where they have been called “Telomeric Associated Sequences” (TASs). Therefore, we decided to use an all-vs-all BLAST comparison of assembled chromosomes to identify putative subtelomeric sequences in *Toxoplasma*. We validated this bioinformatic strategy using the *P. falciparum* 3D7 genome assembly (PlasmoDB v8.2, where TASs were already identified [28]). In *Plasmodium* we observed that similarities among chromosomes were restricted to telomeric and subtelomeric sequences (Additional file 1A), and to regions in the center of the chromosomes 4, 6, 7, 8, and 12 that also contain subtelomeric genes, and are enriched in H3K9me3 a histone mostly restricted to telomeres in *P. falciparum*[30]. We next performed the same all-vs-all BLAST comparison using *Toxoplasma* chromosomal assemblies of the ME49 genome (TgME49), obtained from ToxoDB version 7.3 [3]. As a result, we identified a conserved region of approximately 30 Kb at the end of 9 out of the 14 chromosomes with high identity among them (Figure 1A, Additional file 1B). Because these sequences present the same pattern of sequence similarity observed in *P. falciparum* subtelomeric regions, we denominated them *T. gondii* TAS-like sequences (TgTAS-like or TgTASL). These TAS-like regions were found in *T. gondii* chromosomes Ia, II, III, IV, V, IX, X, XI and XII; with chromosomes III and X containing two such regions each. The similarity among the different TgTASL and their localization can be observed in Figure 1A. In the figure, chromosomes are laid out in a circle, and blocks of sequence similarity longer than 180 bp and with >80% sequence identity are represented with ribbons; those corresponding to TgTASL sequences are highlighted in red. Consequently, black ribbons in Figure 1 correspond to other inter-chromosome repeated sequences, which most of them correspond to the mitochondrial-like repeats (data not shown) [34]. Interestingly, at the end of chromosome XI and the begining of VIIb there are short sequences similar to TgTASL (red links Figure 1A, Additional file 1B). These sequences were not included in our analysis because they were too short, but they could represent TgTAS-like regions that were not correctly assembled. Figure 1 A also shows an additional data track corresponding to the annotated genes in the ME49 genome. A closer inspection of the TgTASL regions shows that they are depleted of genes, with the exception of only one gene located next to one end of the TgTASL region (blue arrows in Figure 1B).

### The putative *Toxoplasma*Telomeric Associated Sequences have a conserved structure

Subtelomeric sequences often display a pattern of DNA repeat blocks, linker regions and sometimes genes. To uncover such patterns in our TgTASL regions, we performed detailed dotplots for the pairwise comparisons of them using Dotter (see Additional file 2), [35]. These plots allowed us to define the limits and organization of each TgTASL region. These limits were arbitrarily restricted to the shared (consensus) sequence present in all TgTAS-like regions. The location of these regions in each chromosome is shown in Table 1. We named each TgTASL based on the chromosome number where it is located (for example TgTASL_Ia is the TgTASL of chromosome Ia). In cases where more than one TgTAS-like was present in a chromosome, we added a letter suffix (“-a”, “-b”, etc.) to reflect this fact (for example in chromosome III there is one TgTASL_III-a near the chromosome end and a second TgTASL_III-b towards the centromere, see Figure 1A, and Table 1). It is important to highlight that there are other genomic sequences that were excluded from our analysis because they are shared among a few chromosomes but do not present a conserved pattern (for example the sequences between TgTASL_III-a and _III-b which are also present at the end of chromosome IX and Ia). Nevertheless, this observation suggests that TgTAS-like regions could be larger than we describe here and/or that other sequences with similar characteristics can be found.

**Table 1:** **Localization of putative TgTASL regions**

ID	chr	ME49	GT1	VEG
TgTASL_Ia	Ia	1–15692 (15691)	1–22843 (22842)	1–25042 (25042)
TgTASL_II	II	2217257–2260968 (43711)	2241754–2265002 (23248)	2245054–2290271 (45217)
TgTASL_III-a	III ^1,2^	2446355–2471000 (24645)	70927–103813 (32886)	9692–53051 (43359)
TgTASL_III-b	III	2291623–2321755 (30132)	2287172–2317147 (29975)	2285863–2316508 (30645)
TgTASL_IV	IV	2179605–2207233 (27628)	2212915–2240672 (27757)	2244426–2273211 (28785)
TgTASL_V	V	100060–130829 (30769)	16–29792 (29776)	432280–461875 (29595)
TgTAS-L_VI	VI^3^	1–2528 (2527) ^*a*^	3642075–3650379 (8304)	1713506–1741000 (27494)
		1–6928 (6927) ^*b*^		
		1–7727 (7726) ^*c*^		
TgTASL_IX	IX	74193–94753 (20560)	116577–138592 (22015)	72421–100418 (27997)
TgTASL_X-a	X	2008–28943 (26935)	1–26609 (26608)	40642–71653 (31011)
TgTASL_X-b	X	65038–90348 (25310)	59949–85376 (25427)	120781–147164 (26383)
TgTASL_XI	XI	634–27485 (26851)	1–29121 (29120)	1–27453 (27453)
TgTASL_XII	XII	24–16153 (16129)	44111–88494 (44383)	55337–111064 (39762)

Chromosome ranges correspond to the start and end coordinates of each TgTAS-like in the corresponding chromosome.

^1^TgTASL_III-a in GT1 strain is present in the scaffold scf_1107000999196.

^2^TgTASL_III-a in VEG strain is present in the scaffold scf_1104442825704.

^3^TgTASL_VI in ME49 strain can be assembled from the contigs: DS984876 (a), DS984831 (b), and DS984825 (c).

**Figure 2:**
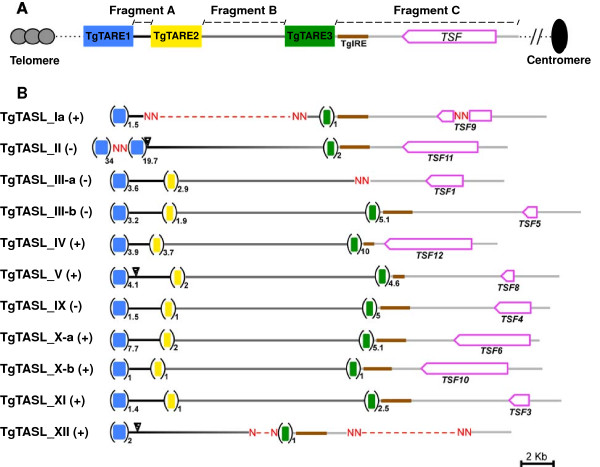
**Internal structure of Telomeric-associated sequences in** ***Toxoplasma*** **.** The figure shows a schematic representation of the internal organization of *Toxoplasma* telomeric-associated sequences - like. **A.** represents the consensus patern TSF = *Toxoplasma* Specific Family [5]; TgTARE = *Toxoplasma* Telomere Asociated Repetitive Elements, TgIRE = *Toxoplasma* Interspersed Repetitive Element [38]. **B.** Scaled representation of each TgTASL. The (+) and (-) next to the TgTASL name, means sense or antisense DNA strand location, respectively. The number of copies of each TgTARE is denoted with a subscript. Triangles point the sequences analyzed by ChIP- qPCR.

Dotplots comparing one TgTAS-like with itself revealed the presence of internal repeats (Additional file 2B). Three blocks containing tandem repeats were identified and named “*T. gondii* Telomeric Associated Repeat Element (TgTARE)” 1 to 3. These repetitive blocks were separated by non-repetitive DNA fragments which we denominated as Fragments A to C, respectively (Figure 2A, Additional file 3A). Each TgTARE was analyzed with Tandem Repeat Finder [36]. In all cases, the tandemly repeated unit was similar but not identical in size and sequence. Their sizes were ∼ 350 bp or ∼ 680 bp for TgTARE1, ∼ 250–300 bp for TgTARE2, and ∼ 250–300 bp for TgTARE3 (Additional file 3B). The identity of these repeats was analyzed by BLAST. Interestingly, TgTARE1 belongs to the satellite DNA Sat350/Sat680 family already described to be near *Toxoplasma* telomeres by Bal31 digestion [37], and also detected in *Neospora caninum*[38]. Another *Toxoplasma* element already described to be present at the end of several chromosomes is TgIRE [12, 39]. This element was found to be part of every TgTASL, located contiguous to the TgTARE3 in Fragment C (Figure 2B, Additional file 3A).

We also inspected annotations and functional genomics data available at the ToxoDB resource. Except for one gene localized at the end of every Fragment C (see Figure 2B, and Additional file 3A), the lack of transcript expression and protein expression data mapped to these regions suggest that TgTASL are mostly silent and/or gene-free DNA. The single gene located at the end of Fragment C is a hypothetical protein annotated as a member of the ‘*Toxoplasma* Specific Family’ (TSF) [5]. Interestingly, the chromosomal location of TSF family members is restricted to the TgTASL region. Based on this observation, we could identify two extra members of this family: TGME49_098060 as TSF11, and TGME49_100990 as TSF12 (Figure 2B, Additional file 3C). All these findings confirm that TgTAS-like regions have a conserved canonical structure.

All TSF genes are apparently transcribed; they have associated EST and RNA-seq data, as well as H3K9ac and H3K4me3 peaks that indicate the presence of an active promoter. In addition, the transcript levels of these genes seem to vary among parasite strains (type I, II, and III), stages (tachyzoite, bradyzoite, sporozoite), and/or cell cycle (Additional file 3C, expression evidence at ToxoDB v7.3). However, only TgTSF8 has evidence of expression at the protein level (mass spectrometry evidence at ToxoDB v7.3). This behavior is similar to the one described for subtelomeric genes. Further studies should be performed to elucidate the role of TSF members.

Comparing TgTASL chromosome coordinates (Table 1) and their DNA strand location (Figure 2), it is clearly evident that these genomic elements have a defined orientation. TgTARE1 is located at the beginning of the TgTASL, whereas the TSF is at the end of the element, being in the forward strand when it is located at the beginning of the chromosome, and in the reverse strand when it is located at the end of it. Therefore, TgTARE1 will be closer to the telomere and TSF toward the centromere. This is true for all TgTASL except for TgTASL_IV and TgTASL_IX, which are in an inverted orientation: they are both at end of the chromsome but in the forward strand. Interestingly, in the current genome assembly (ToxoDB v9.0) the TgTASL_IX is now at the end of chromosome XII in the reverse strand, as observed for the rest of the TgTASL sequences (Additional file 3G); hence, TgTASL_IV is the unique TgTAS-like region with a different orientation.

It is expected that subtelomeres start next to the tract of telomeric repeats. In *Toxoplasma* the TgTASL are located at the ends of chromosomal assemblies, but their proximity to the telomeric repeats is hard to determine because the chromosome ends are not completely assembled. In *Toxoplasma* the telomeric repeat seems to be TTTAGGG. Tracts of this repeat are assembled only at the end of chromosomes Ia, X and XI, and at the beginning of chromosomes III and XI. Only one TgTASL is close to them, TgTASL_XI, which is next to the telomeric repeat (data not shown). In other cases (TgTASL_Ia, _X, and _XII), where telomeric repeats are not assembled, the TgTASL are the first or last sequence region in assembled chromosomes (Table 1, Additional file 3F). Except for TgTASL_IV, all TgTAS-like are within the 6% final portion of each chromosome (Additional file 3F). Therefore, in general, these regions are either next to or in close proximity to telomeres. Recently, more repeat tracts of TTTAGGG have been assembled (ToxoDB v9.0). They can be observed at both ends of chromosomes Ib, III, XI, IV y VIIa, at the beginning of chromosomes VI and VIIb, and at the end of chromosomes Ia, IX y XII. They appear in the forward strand when they are the first chromosomal sequence element, and in the reverse strand when they are the last one. In this assembly, there is another TgTASL close to a telomeric repeat, TgTASL_III-a. There are ∼ 50 Kb between the telomeric repeat and this TgTASL, where there are not annotated genes (Genome Browser in ToxoDB v9.0). Other curious observation is that the telomeric repeat at the end of chromosome IV is not so close to the end, and it is in the forward strand. Interestingly, it is ∼ 60 Kb upstream the TgTASL_IV, which is the unique TgTASL at the end of a chromosome in the forward strand. In addition, the genomic sequence between the telomeric repeat and this TgTASL does not contain any annotated genes, as occur with TgTASL_III-a.

### TgTAS-like regions present a special nucleosomal composition, enriched in heterochromatin markers

**Figure 3:**
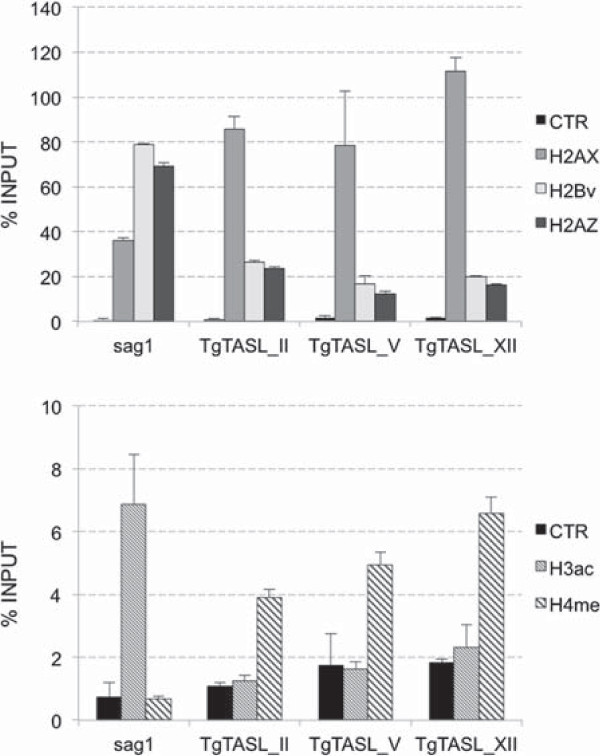
**Nucleosome composition of TgTASL.** Chromatin immunoprecipitation followed by qPCR (ChIP-qPCR) was carried out using anti-H2A. X, –H2A.Z, –H2B.Z, –H3ac, and –H4K20me3 antibodies. A rabbit preimmune antibody was used as negative control (CTR). The amount of TgTASL_II, V, and XII associated to each histone was determined by qPCR, and represented as % Input (where the Input is the 10% of total parasite lysate used per reaction).

Several sequences located within TgTAS-like regions were previously reported to hold characteristics of silent chromatin. Satellite DNA Sat350, (corresponding to TgTARE1) was shown to be enriched in the heterochromatin histone markers H4K20 mono- and tri-methylated [8, 40]. In addition, there is a group of small RNAs identified in *Toxoplasma* that perfectly map to Sat350 repeats [40], probably contributing to their heterochromatin state. On the other hand, we already published that TgIRE, localized in Fragment C, is enriched in the heterochromatin markers H4K20me1 and H2A.X [14]. Almost the rest of Fragment C corresponds to the TSF gene sequence. These genes are rich in H3K4me1 whithin the entire gen body, as it is observed for most genes in *Toxoplasma* (ToxoDB Genome Browser). Based on the availability of these previous data, we decided to analyzed the presence of these markers in sequences within Fragment A by ChIP-qPCR, together with the euchromatic histone markers H3ac, H2A.Z and H2B.Z [7, 12, 13]. The amplified sequences specifically match Fragment A of TgTASL_II, _V and _XII (triangles in Figure 2B); where it was possible to design primers in order to obtain unique and specific products. In all cases, the ChIP-qPCR profile observed was similar, being enriched in heterochromatin markers (Figure 3). The sag1 promoter was used as a control of active chromatin [14]. These data increase the evidence supporting the idea that TgTAS-like regions are indeed silenced domains of the chromosomes, in agreement with the data presented above.

### TgTAS-like regions are conserved in three *T. gondii*evolutionary lineages

Initial studies showed that the majority of *T. gondii* strains isolated in Europe and North America belong to three distinct clonal haplotypes, called types I, II, and III [41]. In this section, the analysis performed with the ME49 (type III) genome data was extended to the genomes of the GT1 (type I) and VEG (type II) strains (data available in ToxoDB). Using the same BLAST strategy to detect similarity among chromosomes, all the TgTAS-like elements detected in ME49 were also detected in these two lineages (Table 1). In addition, we compared all identified TgTASL among strains. In all cases, the TgTASL regions were syntenic (Additional file 4), and the pairwise identity between each TgTASL and its syntenic partners in other strains was >96%. Furthermore, a new TgTASL regions was found in chromosomes VI of the VEG and GT1 strains (Table 1), which was not previously detected in ME49 chromosomal assemblies. We took advantage of the high identity between TAS-like regions in the three lineages, and used TgTASL_VI of GT1 and VEG to perform BLAST searches in ME49 contigs that were left out of the chromosomal assembly. Sequences corresponding to a putative TgTASL_VI could be identified in the ME49 contigs DS984876, DS984831, DS984825 (Table 1). The presence of additional sequences similar to TgTAS-like was examined in contigs and scaffolds, in the three strains: ME49, GT1 and VEG. Several sequences were retrieved, including contigs/scaffolds containing a complete TgTASL (Additional file 3E). These could represent assembly artifacts, or *bona fide* TgTASL regions that could not be accommodated properly in the current assembly. In total, we detected twelve TgTASL regions distributed in 10 chromosomes (Table 1).

Recently, a new *Toxoplasma* ME49 genome assembly was made available in ToxoDB (version 9.0 release, September 2013). Although most of the TgTASL regions are conserved in the two assemblies, some differences could be observed. In the newest version there are two extra TgTASL regions, one at the opposite end of chromosome II, and one in chromosome X, upstream of TgTASL_ X-a (Additional file 3F); in addition to the relocation of TgTASL_IX. Overall, this suggests, together with the sequences found in scaffolds, that there may be additional TgTAS-like sequences. The current draft nature of the *T. gondii* genome currently prevents us from knowing if both chromosome ends are covered with these TAS-like sequences.

Finally, we also investigated the conservation of these TgTAS-like regions in other coccidian genomes available in ToxoDB (*Neospora caninum* and *Eimeria tenella*). BLAST searches using TgTASL sequences as query only revealed matching sequences with low similarity to these genomes. Besides, TgTASL sequences do not present detectable similarity to other Apicomplexan genomes available in EuPathDB. By using the same intra-species all-vs-all chromosomal comparisons used before, we could identify two putative *N. caninum* TAS-like regions in Nc chromosomes VIIa and VIIb, as well as other related DNA fragments (Additional file 3D, Additional file 5). The presence of a conserved structure of repetitive elements in these NcTASL regions were evaluated by pairwise comparisons using Dotter. Dotplots with representative results are shown in Additional file 5. In this analysis it is clear that NcTASL also present a conserved pattern, consisting of two blocks of repeats separated by non- repetitive DNA (first two dotplots of the first two rows in Additional file 5). However, the similarity between NcTASL and TgTASL is extremely low (three last panels of the first two rows in Additional file 5). Consequently, TgTAS-like regions are composed of sequences that are unique to *Toxoplasma*.

### TgTAS-like show a unique bias in trinucleotide composition

Because TgTAS-like regions are depleted of genes and are localized near chromosome ends, we reasoned that they might present a different base composition from the rest of the genome. We thus investigated a number of ways to identify compositional bias in these sequences. For this, we analyzed the nucleotide composition of these regions using different metrics, which are all independent of linear sequence similarity. The trinucleotide composition of TgTAS-like sequences was used in our case to identify diagnostic biases. Our measure of compositional bias simultaneously considered all 64 trinucleotides across a given sequence using a multivariate statistical technique (Correspondence Analysis, see Methods).

Correspondence Analysis (CA) is a powerful statistical technique to explore high-dimensional categorical data. The trinucleotide composition of a DNA sequence is one such dataset; with each dimension/column measuring the count of a different trinucleotide (there are 64 trinucleotides/dimensions) in different genomic fragments (rows). The aim in a correspondence analysis is to identify trends in the variation of data and associations among datapoints. CA has been successfully applied to the study of synonymous codon usage bias associated with high levels of expression [42–45]. Using this technique, it is possible to summarize and explore most of the 64-dimensional information using only a few representative axes. This allows visual representations in 2- dimensional plots (also known as “CA maps”) that capture the most influential trends that define the compositional bias.

**Figure 4:**
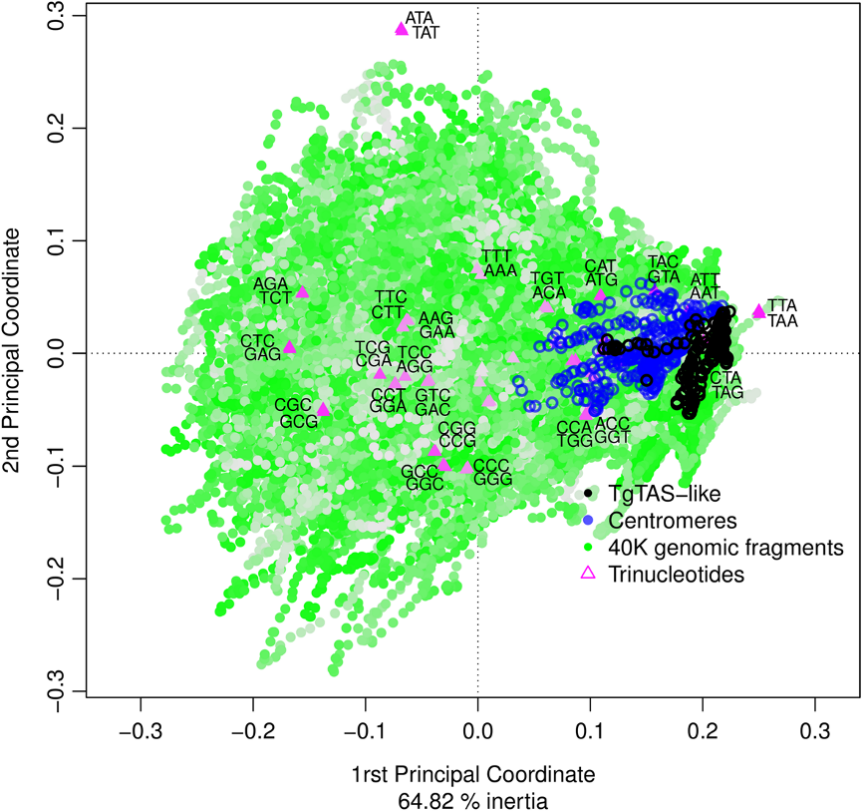
**Symmetric map (biplot) of trinucleotides and genomic fragments from a Factorial Correspondence Analysis (CA).** Correspondence analysis was performed as described in Methods. The 1st and 2nd coordinates plotted in the map represent 65% of the total inertia. Data points that are close to the origin (0,0) have no compositional bias (homogeneous usage) while points that move away from the origin represent larger deviations. In the plot, data points corresponding to genomic fragments (N = 40 Kb) are shown in green, and are overlayed with data points corresponding to centromeric sequences in blue), data points corresponding to TgTAS-like sequences (in black), along with the 64 trinucleotides (in magenta). Opacity of colours is related to how well each point (its inertia) is represented in this 2 dimensional subspace. Plot interpretation: fragments (dots) that are close in the plot have similar trinucleotide composition, and trinucleotides (triangles) which are close to each other frequently appear in the same fragments. Distances between dots and triangles have no meaning, however, the directions of dots and triangles from the origin (0,0) are meaningful (e.g. fragments on the right of the origin use more TAA, CTA, ATT (and their reverse complements TTA, TAG, AAT) and less AGA, CTC and CGC (and reverse complements TCT, GAG and GCG), whereas the opposite is true for the fragments lying on the left of the origin).

**Figure 5:**
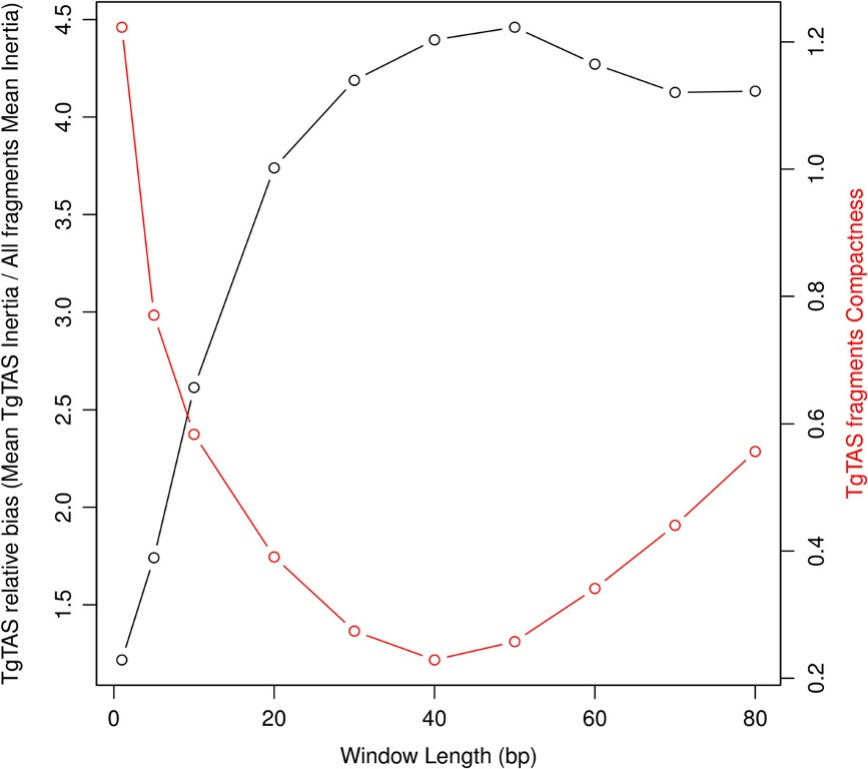
**Strength of the compositional bias signal of TgTASL sequences over different lengths of genomic fragments.** The curve in black shows the bias ratio of TgTASL-containing genomic fragments relative to the compositional bias of all genomic fragments at a given window length. For example, at 1Kbp fragments, TgTASL containing fragments show a relative bias of 1, ie. there is no TgTASL specific bias, while there is a peak at 50Kbp, with a relative bias of ∼4.5 (ie, TgTASL have on average ∼4.5 times more trinucleotide bias compared to all the genomic fragments). The curve in red shows the divergence in trinucleotide composition of TgTAS-like containing fragments. At 40Kbp the divergence presents a minimum, ie the TgTASL fragments are highly compact.

We performed a CA of trinucleotide composition of the *Toxoplasma* ME49 genome, using different fragments lengths (see Methods). This analysis allowed us to compare the trinucleotide composition of TgTASL fragments to that of the genome. As a result of this analysis we found that TgTASL regions display a unique trinucleotide composition. They are highly enriched in TAA, TAG, ATT (and their reverse complements TTA, CTA, AAT), moderately enriched in other trinucleotides such as TAG/CTA, ATG/CAT, TGA/TCA, while depleted of AGA/TCT, CTC/GAG and CGC/GCG (Figure 4). In this compositional signature, both the enrichment and the under-representation of some trinucleotides is important to explain TgTASL composition preferences. This compositional bias was not detected at 1 Kb nucleotide fragments, but it was significant at 10 Kb, and peaked at 40–50 Kb resolution (Figure 5 left axis, relative bias), which is the average size of TgTASL regions. For larger window sizes, the composition of different TgTAS-like containing fragments begins to diverge due to mixture of different genomic contexts within the same window (Figure 5, right axis, TgTASL fragments compactness). CA maps for all window lengths are shown in Additional file 6. Interestingly, a similar trinucleotide bias was also observed in centromeres (Figure 4, Additional file 6), which are also gene-depleted genomic regions [26], but without any similarity at sequence level among them and/or with TgTAS-like regions.

In the CA analysis mentioned above (window size = 40 Kb), the major compositional trend, represented in the 1st principal coordinate (horizontal axis of Figure 4), explains 45% of the information on the variability of trinucleotide frequencies. This means that CA is effectively summarizing high-dimensional data, due to the presence of strong trends (biases). The second trend, represented as the 2nd principal coordinate (vertical axis in Figure 4), which is independent to the 1st one (CA produces orthogonal axes by design), is lead by one trinucleotide pair (ATA, TAT). Upon further inspection, we found that this trend is due to the presence of sequences with long TpA dinucleotide repeats, which are not highly represented in TgTAS-like regions. The relative contribution of each trinucleotide to the 1rst and 2nd Principal Coordinates are listed in Additional file 3H.

### The unique trinucleotide bias of TgTAS-like sequences is shared with other gene-poor genomic regions in the *T. gondii*genome

**Figure 6:**
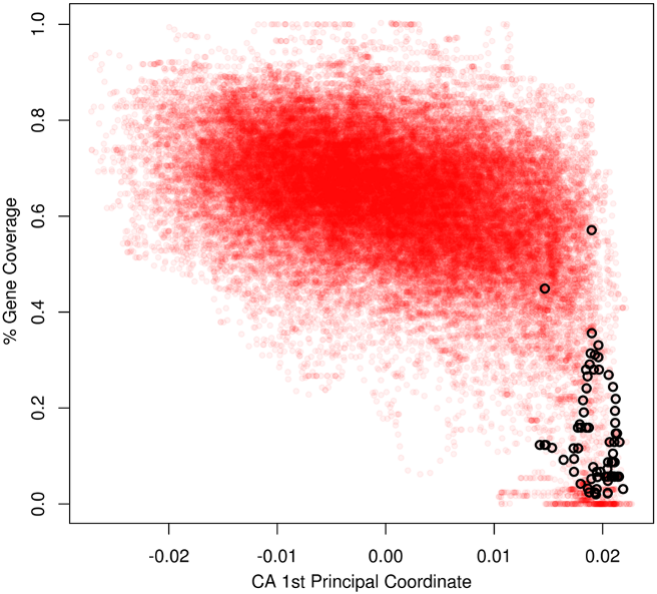
**Trinucleotide bias vs gene density.** Scatterplot of trinucleotide compositional bias (Correspondence analysis 1rst Principal Coordinate) at 40Kb vs. gene coverage (ie, proportion of nucleotides in a genomic fragment, being part of a gene) for all 40K genomic fragments, overlapped by 39K. TgTAS-like associated fragments are painted black. A strong non-linear relation can be appreciated between the variables, where low gene density regions (including gene-depleted regions) present a particular trinucleotide composition (high 1PC). Also, there is a negative linear component relating both variables (Pearson’s correlation coefficient –0.445, p-value < 10 ^-16^).

We have identified many non-TgTAS-like regions that show a trinucleotide composition bias that is similar to that observed in TgTAS-like regions (Figure 4, previous section). A group of such non-TgTASL regions are the centromeric sequences, which are also gene-poor regions. To further explore the observed relationship between trinucleotide bias and coding potential we calculated a gene density index across the genome, and compared this to the trinculeotide bias (1PC in Figure 4). The first principal coordinate was negatively correlated to gene density across the genome (Pearson’s correlation coef. –0.445, P-value <10*E*^-16^). Genomic fragments with low 1PC values are exclusively gene-rich while high 1PC values are associated to gene-poor or gene-depleted regions, such as TgTAS-like regions and centromeres (Figure 6). This is also consistent with the observed over-representation of stop codons in sequences with high 1PC values. Genomic regions with similar TgTAS-like compositional bias were identified at every chromosome end (Additional file 7), which might correspond to other subtelomeric sequences. However, other regions with a similar compositional bias were also detected across all chromosomes, probably denoting gene-poor regions and/or heterochromatin, which should be confirmed experimentally.

## Discussion

Telomeric and subtelomeric heterochromatin differs from standard heterochromatin on several aspects including its sequence, nucleosome composition, and nature of binding factors. Even though these three components vary between species, telomeric function is conserved [17]. Here we have identified and characterized a number of *Toxoplasma* TAS-like regions, which are localized near several chromosome ends, and are specific for *T. gondii*. They have features similar to those observed in other subtelomeric sequences like blocks of repeats, stress-asociated genes, and a heterochromatin-like structure, based on the presence of informative histones and histone post-translational modifications.

Even though the *T. gondii* genome was recently further sequenced and re-assembled, the subtelomeres of this important parasite were not identified or characterized. Telomeres and subtelomeric sequences are noticeably hard to assemble when using shotgun sequencing. This is because the telomeric repeat is identical in all chromosomes and therefore cannot be used to distinguish one telomere from another. Furthermore, they are not expected to be well represented in the Bacterial Artificial Chromosome (BAC) libraries used to guide the scaffolding and chromosomal assembly of draft genomes, due to the size-selection of recombinant DNA clones and the natural genomic instability of these regions. Finally, they provide large similarity segments that are rich in repetitive sequences, which are the cause of frequent assembly errors. Using the current chromosomal assemblies, we mapped TgTAS-like sequences to a conserved region of 30 Kb located at the end of most chromosomes. We defined the extent of these regions based on the consensus TgTAS-like structure present in most chromosomes. However, it is reasonable to consider that the actual size of each TgTASL may be larger. In this regard, the fact that the trinucleotide compositional bias peaks at 40-50Kb provides an independent estimate for the extent of these regions.

In all cases, the identified TgTASL regions displayed a similar structural pattern, consisting of three telomeric associated repeats (TgTARE 1-3), with a single member of the *Toxoplasma* Specific Family, separated by silenced DNA. These TgTASL regions have a clear chromosomal orientation, with the TgTARE1 towards the telomere, and the TSF gene towards the centromere; similar to *P. falciparum* TASs [28]. Nevertheless, several exceptions were detected in the ME49 genome that still remain in the new genome assembly (ToxoDB 9.0). In chromosomes III and X there are two consecutive TgTASL regions, and in chromosome IV, the TgTASL_IV is inverted (its TgTARE1 is towards the centromere and the TSF gene towards the telomere). These unusual features, which so far are unique to *Toxoplasma* genomes, as well as the presence of TAS-like regions at only one end of the chromosomes, could be explained by simple chromosomal rearrangements, probably with functional consequences. However, they could also be explained by artifacts of the genome sequencing and/or assembly. Several observations support this last possibility: changes in the chromosome location and number observed between TgTAS-like regions in two different genome assemblies (Additional file 3F); the relocation of TgTASL_IX which is now located at the end of chromosome XII with the expected orientation; and the absence of BAC-end sequences connecting TgTASL regions with the rest of the assembled chromosome, as in the case of TgTASL_IV (ToxoDB Genome Browser; see also Figure 4 in Khan *et al.* publication [4]). Therefore, further studies should be performed to shed light on these issues. Also future genome assemblies should pay special attention to the quality of the assemblies near chromosome ends.

From human to yeast, the shelterin protein complex binds DNA telomeric repeats, and subtelomeric sequences are silenced as evidenced by the presence of telltale heterochromatin epigenetic marks such as histones H3K9me3 and H4K20me3, methylated DNA, and heterochromatin protein 1 (HP1) [46, 47]. Telomeric and subtelomeric sequences are transcribed in a telomeric repeat-containing RNA (TERRA), which regulates the telomere function [47]. Some telomeric binding factors have been described in *P. falciparum* including PfSir2a, a sirtuin that may be important to maintain DNA as heterochromatin [31, 48]; PfOrc1 that binds to telomeres and TARE-3, and may be involved in forming the T-loop structure that prevents fusion between chromosome ends [48]; and PfSIP2 (ApiAP2 family member) that binds to TARE2, TARE3 and the upsB promoter of a var gene, and colocalizes with PfHP1 that binds H3K9me3, suggesting that both proteins participate in the assembly of telomeric heterochromatin [49]. With the exception of TgChromo1 [10], none of these proteins have been characterized to date in *Toxoplasma*, despite the fact that all of them have orthologs. TgChromo1 is the HP1 chromobox homologue. A number of studies focused on this protein provide additional evidence that TgTAS-like regions are embedded in a typical heterochromatin environment. Although TgChromo1 is mainly associated with pericentromeric heterochromatin, IFA-FISH experiments have shown that TgChromo1 is also in close proximity to repeats present at the end of chromosome Ia and IX at the nuclear periphery [10]. Interestingly, the chromosome Ia probe used by Gissot *et al.*[10] corresponds to TgTARE1, suggesting that TgTASL elements could be located at the nuclear periphery. In a separate set of ChIP- qPCR experiments, Braun *et al.* detected an enrichment of H4K20me1 and H3K9me1 heterochromatin marks associated with the Sat350 repetitive element (which would be TgTARE1) [40]. We also revealed the enrichment of H4K20me1 and H2A.X (another silencing-associated histone in *T. gondii*[14]) in fragment A from three TgTASL, and also in TgIRE [14]. In addition, Braun *et al.* uncovered the presence of repeat-associated siRNAs in *Toxoplasma*, which map to Sat350 (TgTARE1) and Sat529a [40]. It remains to be seen if these satellite-associated RNAs are involved in heterochromatin formation and/or in the regulation of longer telomeric noncoding RNA resembling the TERRA RNA from other organisms [50].

Genes that are close to subtelomeric regions have been implicated in a wide array of stress responses or niche adaptive roles [17]. This is also true for a number of important human pathogens, including *T. brucei* and *T. cruzi*, *L. major*, and *P. falciparum*, where so called contingency gene families are located embedded within or just next to the subtelomeric repeats. *Toxoplasma* contains several distinct, coccidian-specific multicopy gene families throughout its genome, including those that encode the SRS, ROPK, and SUSA proteins [51–53]. Recently, a bioinformatics study showed that 60 out of the 144 ME49 SRS genes (42%) are located in subtelomeric sites [54]. However, none of these genes are near TgTAS-like regions in the current genome assembly. We only detected one *Toxoplasma* specific family (TSF) member [5]. These TSF members were also found in VEG and GT1 *T. gondii* strains. Judging by the sequence, length, and number of TM domains, this family presents a high diversity among their members (Additional file 7C). Moreover, such diversity extends to their expression profiles, as the apparent expression of each TSF gene varies among different strains, life cycle stages, and/or throughout the cell cycle (Additional file 3C). Notwithstanding this, there is mass spectrometry evidence for only one TSF member, according with the experimental data available in ToxoDB version 7.3. Although the function of TSF genes is unknown, their expression profiles resemble those of tightly regulated genes associated to parasite adaptation to the environment (differences between species), or virulence (differences between strain). Hence, they could be proposed as *Toxoplasma* stress and/or contingency response genes. Notably, recent RNAseq experiments revealed some discrepancies between transcripts and annotated genes, and supported the existence of new genes and/or putative non-coding RNAs ([55], and RNAseq evidences in ToxoDB v9.0). Some of these RNAseq transcripts can be detected within several TgTASL, suggesting there might be more genes present in these regions.

The trinucleotide composition of TgTASL DNA displays a strong bias when analyzing genomic fragments greater than ∼5 Kb. However this bias it not evident below this size, indicating that this is a long-range bias. The major compositional trend is associated with the relatively high frequency of stop codons in these regions, in correlation with the absence of genes. However, the trinucleotides TAA, TAG and TGA (together with their reverse complements) which are read as Stop codons within coding sequences, only contribute to 17.4% of this major trend. Other trinucleotides, not particularly associated with non-coding sequences, such as the pairs CTC/GAG, AGA/TCT, CGC/GCG, AAT/ATT, GTA/TAC have larger contributions to this major trend (in Additional file 3H). For example, the trinucleotide pairs AAT/ATT (enriched) and AGA/TCT (depleted) together represent 19.6% of the bias in the major trend, and show a clustering pattern of TgTAS-like and centromeric sequences similar to the one observed for the stop codons (Additional file 8).

Interestingly, the second most relevant trend at ∼40 Kb is led by a single trinucleotide pair ATA/TAT. These trinucleotides also represent the major trend (1PC) at shorter fragments (1Kb). Although, there are other AT-rich trinucleotides such as ATA/TAT and TTA/TAA, they define two major independent trends, respectively. The trinucleotides ATA/TAT (but not TTA/TAA), which also govern the compositional bias at shorter window sizes, are part of microsatellite-like sequences (TpA dinucleotide repeats). These TpA tracts have the lowest base stacking energy, and therefore the greatest flexibility for unwinding the DNA (like in the TATA box); consecuently, they provide interesting functional features to these genomic territories in *T. gondii*[56].

All observed trinucleotide frequencies were approximately identical to the frequency of their reverse-complementary trinucleotides in the *T. gondii* genome (Figure 4). These correlations between a trinucleotide and its reverse complement were evident even at windows of size 5 Kb, and strongly increase for larger fragments. We constructed these CA maps with trinucleotide counts obtained from a single-strand of each chromosome to show an important consequence of this fact: TgTAS-like regions located in the positive strand cluster together with negative-strand TAS (i.e. their trincucleotide compositions are highly similar), even though their sequences are completely different (they are reverse-complements). This forward/reverse- complement symmetry of trinucleotide frequencies is part of a more general property: Chargaff’s second parity rule. This rule states that within a single strand of double-stranded DNA, each oligonucleotide occurs with approximately the same frequency as its reverse-complement [57]. This observation has been verified for “sufficiently long” (>100 Kb) genomic sequences, in a wide range of sequenced genomes of bacteria and eukaryotes [58]. Here we confirm that this is also true within the TgTAS- like regions of ∼40 Kb in *T. gondii*.

Finally, it is also noteworthy that the major trend (1PC) in trinucleotide composition allows the separation of TgTAS-like regions from the rest of the genome. This essentially means that TgTASL represent some of the most peculiar regions of the genome when looked at the level of trinucleotide composition. Other genomic regions that share the same compositional bias are centromeric fragments, most of the chromosome ends in the current assembly, as well as other chromosomal internal regions (see Additional file 7). We propose that this compositional bias may be associated with a more general property of these regions, such as their preferred chromatin state. However, further studies will need to be performed to elucidate the functional features shared by all these regions in *T. gondii*.

## Conclusions

In this work we have identified and described a number of chromosomal regions, that present a special long-range compositional bias, are gene-poor, and enriched in repeats and in heterochromatin-associated histones. These genomic domains are usually present in subtelomeric and subtelomeric-like regions. In addition, this long range compositional bias was also detected in other *Toxoplasma* gene-poor genomic regions that do not share any sequence similarity among them. Future studies are planned to further study the protein(s) associated to these TgTAS-like regions as well as the role of these elements in *Toxoplasma* biology.

## Methods

### Sequence resource, analysis and representation

The genome sequence, coding sequences, and gene annotations were downloaded from ToxoDB version 7.3 (www.toxodb.org). Some results were compared with the current version of ToxoDB v9.0. *P. falciparum* 3D7 genome assembly was download from PlasmoDB v8.2 (www.plasmodb.org). Comparative genomics were performed using BLAST version 2.2.25 obtained from the NCBI [59], and WebACT [60]. BLAST results were analyzed and visualized with the Artemis Comparative Tool (ACT, http://www.sanger.ac.uk/Software/ACT) [61]. Circular chromosome layouts were made with circos (http://circos.ca/) [62]. The size and repeats of TgTASL regions were determined by visual inspection of chromosomal dotplots generated with Dotter (http://sonnhammer.sbc.su.se/Dotter.html) [35]. To facilitate the pairwise comparison of all chromosomes, we produced a dotplot comparing a multifasta file containing all chromosome fragments encompassing TgTASL, whose size was estimated using ACT.

### ChIP-qPCR experiments

ChIP and qPCR were performed as described in Dalmasso *et al.*[14]. Briefly, 1 x 10^7^ tachyzoites were used per reaction. The 10% of total lysate was used as input. The antibodies used to perform the immunoprecipitation were: *α*-H2A.X, *α*-H2A.Z, and *α*-H2Bv polyclonal antibodies previously generated in the laboratory [12, 14]; *α*-H3ac (Upstate 17-615), and *α*-H4K20me1 (Abcam ab9051). The specific primers used to amplify the TgTAS-like are listed in Additional file 3G. They were designed to specific amplify each TgTASL, after sequence alignment among them. Sag1 promoter was used to represent a transcriptionally active chromatin region [14]. Three independent experiments were performed.

### Trinucleotide Correspondence Analysis (CA)

*T. gondii* ME49 chromosomes (ToxoDB version 7.3) were scanned through overlapping windows of varying length *L*, by 1 Kb nucleotide steps (where *L* ranged from 1 Kb to 80 Kb). A number of contigency tables of size 64 x *L* were built by counting the number of each trinculeotide in every genomic fragment of length *L*. CA is an exploratory multivariate statistical technique used to decompose the contingency table variability and extract coordinates that explain major trends in the inertia of the table [63]. Inertia is defined as the total Pearson’s Chi- Square of the 2-way table divided by the total sum and can be interpreted as a global measure of the data’s deviation from expected values under the hypothesis of homogeneity (where all genomic fragments would have similar trinucleotide profiles). Correspondence Analysis was performed using the R package CA ([63]). CA plots shown in Additional file 6 are symmetric maps displaying the 2 principal dimensions with most of the trinucleotide variability (rows and columns in principal coordinates). To determine trinucleotide bias associated with TgTAS-like regions, we considered genome fragments containing complete TgTASL regions as well as those fragments where the annotated TgTASL region would cover >80% of the window length. Average deviation of these fragments (*i.e.* TgTAS-like trinucleotide bias, or inertia in the CA theory) from the origin (position representing the expected composition under homogeneity of trinucleotide frequencies) was calculated, and then divided by the average deviation of all genomic fragments to derive a measure of TgTASL-specific bias. This was repeated for all window lengths (Figure 5, left axis). The compactness of TgTAS- like fragments was evaluated by measuring disimilarity in trinucleotide space, calculated as the mean of all TgTAS-like inter-fragments distances relative to the mean inter fragments distances considering all genomic fragments (Figure 5, right axis). To explore the relation between trinucleotide bias (at 40 Kb window length) and gene density, we calculated a gene coverage index defined as the proportion of bases covered by gene annotations (CDS, gene annotation from ToxoDB 01/2012). The gene coverage index was plotted against a 40 Kb trinucleotide bias index, which is simply the first principal coordinate of the CA.

## Electronic supplementary material

1: **Pairwise comparisons between chromsomes.** ACT visualization of chromosome similarities. The representation only shows the similarities between each chromosome (double grey lines, with coordinates in bp) and the contiguous chromosome, above or below [61]. Links in red genomic are genomic segments on forward strand, and in blue genomic segment on reverse strand. **A**. All *P. falciparum* chromosomes, not being filtered. **B**. *T. gondii* chromosomes containing TgTASL sequences (green box with a dashed line). The asterisks point to short TgTASL sequences present in other chromosomal regions. Sequences were filtered to be at least 180 bp long. (PDF 160 KB)

Additional file 2: **All-vs-all dotplot comparisons of TgTAS-like regions.** Dotplots were generated from a multifasta file containing all TgTAS- like regions. The green lines separate each TgTASL. **A.** The complete dotplot of all TgTAS-like regions in the ME49 strain. The axes on the top and left show the size in Kb, whereas the ones on the bottom and the right shows the name of each TgTASL being compared. **B.** Schematic representation of the dotplot analysis, using part of a row of TgTASL_IV. In the first panel the TgTASL_IV region was compared with itself, clearly showing the three blocks of repeats (TgTARE 1 to 3) being represented by several lines (one per repeat). In the rest of the panels TgTASL_IV was compared with TgTASL_V to _XII showing similarities among these TgTASL regions. Missing sequences, insertions and deletions are visible as gaps in the sequence comparison, for example in the last panel, where a run of Ns connecting two contigs in TgTASL_XII appears as a large gap in the dotplot. (PDF 1 MB)

Additional file 3: **Supplementary tables.** This file contains a number of supplementary tables referenced in the text, or that provide additional support/material for the paper. Each table is contained within a separate spreadsheet in the file: AdditionalFile3.xlsx. A: Chromosomal locations of TgTAS and flanking TSF genes; B: Characteristics of *T. gondii* Telomere-associated repetitive elements - like (TgTAREL); C: List of TSF genes retrieved from ToxoDB v7.3, and their relative expression; D: List of putative *Neospora caninum* TAS -like (NcTAS); E: List of contigs and scaffolds with similarity to TgTASL; F: Comparison of TgTASL between ToxoDB versions 7.3 and 9.0 G: List of oligonucleotide primers used to amplify TgTASL regions. H: Table of compositional bias contribution per trinucleotide. (XLSX 2 MB)

Additional file 4: **Comparative genomics of different** ***T. gondii*** **strains.** Chromosomal similarity visualizations using ACT. BLASTN similarities across chromosomes are shown as red (forward strand), or blue (reverse) segments. Similarity between TgTAS regions is shown with yellow segments. The figure shows a comparative genomics analysis of all chromosomes containing TgTASL in three *T. gondii* strains: GT1 (type I), ME49 (type II) and VEG (type III). The asterisk next to the ME49 chromosome VI is indicating that at the end of the chromosome there are three additional contigs (light blue) containing sequences similar to TgTASL_VI in the GT1 and VEG strains. (PDF 544 KB)

Additional file 5: **Dotplot of** ***Neospora caninum*** **vs** ***T. gondii*** **TAS-like sequences.** The presence of conserved patterns in the NcTASL, and their lack of similarity against TgTASL, were evaluated by all-vs-all pairwise comparison using Dotter. The dotplot includes the 2 putative NcTASL and 3 representative TgTASL. (PDF 159 KB)

Additional file 6: **Correspondence analysis Maps of genomic fragments of 1 to 80 Kb.** This supplementary file contains symmetric biplots similar to those in Figure 4. The PDF file contains a succession of maps obtained for increasing genomic window sizes. (PDF 5 MB)

Additional file 7: **Visualization of TgTAS-like compositional bias along** ***Toxoplasma*** **chromosomes.** A schematic representation of chromosomes is depicted where the major trend in trinucleotide compositional bias (first principal coordinate at N = 40 Kb) is encoded with a color gradient going from cyan (negative values in 1st coordinate, see Figure 4) to magenta (positive values in 1st coordinate), and passing through white (zero, no bias). The position of TgTAS-like and centromeric regions are marked with black and blue boxes, respectively. (TIFF 1 MB)

Additional file 8: **Example scatterplots of trinucleotide abundance.** These plots show the distribution of all 40 Kb genomic fragments according to trinucleotide counts of selected trinucleotides. **A.** This panel shows a symetric biplot for two pairs of trinucleotides: TAA/TTA and TAG/CTA, which are read as STOP codons in coding sequences. These contribute with a 12.7% of the major bias trend when considering all trinucleotides (see text). **B.** This panel shows a symetric biplot for two other influent trinucleotides ATT/AAT and TCT/AGA, contributing with a 19% of the major trend. The two axes show the number of trinucleotide counts in a window of 40 Kb. In both plots the fragments containing TgTAS-like regions are displayed in black and those containing centromeric fragments in blue. (PDF 829 KB)

## References

[1] Tenter AM, Heckeroth AR, Weiss LM (2000). **Toxoplasma gondii: from animals to humans**. Int J Parasitol.

[2] Dubey JP, Sundar N, Hill D, Velmurugan GV, Bandini LA, Kwok OCH, Majumdar D, Su C (2008). **High prevalence and abundant atypical genotypes of*****Toxoplasma gondii*****isolated from lambs destined for human consumption in the USA**. Int J Parasitol.

[3] Gajria B, Bahl A, Brestelli J, Dommer J, Fischer S, Gao X, Heiges M, Iodice J, Kissinger JC, Mackey AJ, Pinney DF, Roos DS, Stoeckert JCJ, Wang H, Brunk BP (2008). **ToxoDB: an integrated*****Toxoplasma gondii*****database resource**. Nucleic Acids Res.

[4] Khan A, Taylor S, Su C, Mackey AJ, Boyle J, Cole R, Glover D, Tang K, Paulsen IT, Berriman M, Boothroyd JC, Pfefferkorn ER, Dubey JP, Ajioka JW, Roos DS, Wootton JC, Sibley LD (2005). **Composite genome map and recombination parameters derived from three archetypal lineages of*****Toxoplasma gondii***. Nucleic Acids Res.

[5] Reid AJ, Vermont SJ, Cotton JA, Harris D, Hill-Cawthorne GA, Konen-Waisman S, Latham SM, Mourier T, Norton R, Quail MA, Sanders M, Shanmugam D, Sohal A, Wasmuth JD, Brunk B, Grigg ME, Howard JC, Parkinson J, Roos DS, Trees AJ, Berriman M, Pain A, Wastling JM (2012). **Comparative genomics of the apicomplexan parasites*****Toxoplasma gondii*****and*****Neospora caninum*****: Coccidia differing in host range and transmission strategy**. PLoS Pathog.

[6] Strahl BD, Allis CD (2000). **The language of covalent histone modifications**. Nature.

[7] Gissot M, Kelly KA, Ajioka JW, Greally JM, Kim K (2007). **Epigenomic modifications predict active promoters and gene structure in*****Toxoplasma gondii***. PLoS Pathog.

[8] Sautel CF, Cannella D, Bastien O, Kieffer S, Aldebert D, Garin J, Tardieux I, Belrhali H, Hakimi MA (2007). **SET8-mediated methylations of histone H4 lysine 20 mark silent heterochromatic domains in apicomplexan genomes**. Mol Cell Biol.

[9] Bougdour A, Braun L, Cannella D, Hakimi M-A (2010). **Chromatin modifications: implications in the regulation of gene expression in*****Toxoplasma gondii***. Cell Microbiol.

[10] Gissot M, Walker R, Delhaye S, Huot L, Hot D, Tomavo S (2012). ***Toxoplasma gondii*****chromodomain protein 1 binds to heterochromatin and colocalises with centromeres and telomeres at the nuclear periphery**. PLoS One.

[11] Malik HS, Henikoff S (2003). **Phylogenomics of the nucleosome**. Nat Struct Biol.

[12] Dalmasso MC, Echeverria PC, Zappia MP, Hellman U, Dubremetz JF, Angel SO (2006). ***Toxoplasma gondii*****has two lineages of histones 2b (H2B) with different expression profiles**. Mol Biochem Parasitol.

[13] Talbert PB, Ahmad K, Almouzni G, Ausio J, Berger F, Bhalla PL, Bonner WM, Cande WZ, Chadwick BP, Chan SW, Cross GA, Cui L, Dimitrov SI, Doenecke D, Eirin-Lopez JM, Gorovsky MA, Hake SB, Hamkalo BA, Holec S, Jacobsen SE, Kamieniarz K, Khochbin S, Ladurner AG, Landsman D, Latham JA, Loppin B, Malik HS, Marzluff WF, Pehrson JR, Postberg J (2012). **A unified phylogeny-based nomenclature for histone variants**. Epigenet Chromatin.

[14] Dalmasso MC, Onyango DO, Naguleswaran A, Sullivan WJ, Angel SO (2009). ***Toxoplasma*****H2A variants reveal novel insights into nucleosome composition and functions for this histone family**. J Mol Biol.

[15] Verdaasdonk JS, Bloom K (2011). **Centromeres: unique chromatin structures that drive chromosome segregation**. Nat Rev Mol Cell Biol.

[16] Henikoff S, Dalal Y (2005). **Centromeric chromatin: what makes it unique?**. Curr Opin Genet Dev.

[17] Ottaviani A, Gilson E, Magdinier F (2008). **Telomeric position effect: from the yeast paradigm to human pathologies?**. Biochimie.

[18] Pryde FE, Louis EJ (1999). **Limitations of silencing at native yeast telomeres**. EMBO J.

[19] Raghuraman MK, Winzeler EA, Collingwood D, Hunt S, Wodicka L, Conway A, Lockhart DJ, Davis RW, Brewer BJ, Fangman WL (2001). **Replication dynamics of the yeast genome**. Science.

[20] Arnoult N, Schluth-Bolard C, Letessier A, Drascovic I, Bouarich-Bourimi R, Campisi J, Kim S-H, Boussouar A, Ottaviani A, Magdinier F, Gilson E, Londoño-Vallejo A (2010). **Replication timing of human telomeres is chromosome arm-specific, influenced by subtelomeric structures and connected to nuclear localization**. PLoS Genet.

[21] Zappulla DC, Sternglanz R, Leatherwood J (2002). **Control of replication timing by a transcriptional silencer**. Curr Biol.

[22] Ofir R, Wong AC, McDermid HE, Skorecki KL, Selig S (1999). **Position effect of human telomeric repeats on replication timing**. Proc Natl Acad Sci USA.

[23] Ottaviani A, Schluth-Bolard C, Rival-Gervier S, Boussouar A, Rondier D, Foerster AM, Morere J, Bauwens S, Gazzo S, Callet-Bauchu E, Gilson E, Magdinier F (2009). **Identification of a perinuclear positioning element in human subtelomeres that requires A-type lamins and CTCF**. EMBO J.

[24] Barry JD, Ginger ML, Burton P, McCulloch R (2003). **Why are parasite contingency genes often associated with telomeres?**. Int J Parasitol.

[25] Saksouk N, Bhatti MM, Kieffer S, Smith AT, Musset K, Garin J, Sullivan JWJ, Cesbron-Delauw MF, Hakimi MA (2005). **Histone-modifying complexes regulate gene expression pertinent to the differentiation of the protozoan parasite*****Toxoplasma gondii***. Mol Cell Biol.

[26] Brooks CF, Francia ME, Gissot M, Croken MM, Kim K, Striepen B (2011). ***Toxoplasma gondii*****sequesters centromeres to a specific nuclear region throughout the cell cycle**. Proc Natl Acad Sci USA.

[27] Figueiredo LM, Pirrit LA, Scherf A (2000). **Genomic organisation and chromatin structure of*****Plasmodium falciparum*****chromosome ends**. Mol Biochem Parasitol.

[28] Scherf A, Figueiredo LM, Freitas-Junior LH (2001). **Plasmodium telomeres: a pathogen’s perspective**. Curr Opin Microbiol.

[29] Hernandez-Rivas R, Perez-Toledo K, Herrera Solorio AM, Delgadillo DM, Vargas M (2010). **Telomeric heterochromatin in*****Plasmodium falciparum***. J Biomed Biotechnol.

[30] Lopez-Rubio JJ, Mancio-Silva L, Scherf A (2009). **Genome-wide analysis of heterochromatin associates clonally variant gene regulation with perinuclear repressive centers in malaria parasites**. Cell Host Microbe.

[31] Freitas-Junior LH, Hernandez-Rivas R, Ralph SA, Montiel-Condado D, Ruvalcaba-Salazar OK, Rojas-Meza AP, Mancio-Silva L, Leal-Silvestre RJ, Gontijo AM, Shorte S, Scherf A (2005). **Telomeric heterochromatin propagation and histone acetylation control mutually exclusive expression of antigenic variation genes in malaria parasites**. Cell.

[32] Mefford HC, Linardopoulou E, Coil D, van den Engh G, Trask BJ (2001). **Comparative sequencing of a multicopy subtelomeric region containing olfactory receptor genes reveals multiple interactions between non-homologous chromosomes**. Hum Mol Genet.

[33] Mefford HC, Trask BJ (2002). **The complex structure and dynamic evolution of human subtelomeres**. Nat Rev Genet.

[34] Ossorio PN, Sibley LD, Boothroyd JC (1991). **Mitochondrial-like DNA sequences flanked by direct and inverted repeats in the nuclear genome of*****Toxoplasma gondii***. J Mol Biol.

[35] Sonnhammer EL, Durbin R (1995). **A dot-matrix program with dynamic threshold control suited for genomic DNA and protein sequence analysis**. Gene.

[36] Benson G (1999). **Tandem repeats finder: a program to analyze DNA sequences**. Nucleic Acids Res.

[37] Matrajt M, Angel SO, Pszenny V, Guarnera E, Roos DS, Garberi JC (1999). **Arrays of repetitive DNA elements in the largest chromosomes of*****Toxoplasma gondii***. Genome.

[38] Clemente M, de Miguel N, Lia VV, Matrajt M, Angel SO (2004). **Structure analysis of two*****Toxoplasma gondii*****and*****Neospora caninum*****satellite DNA families and evolution of their common monomeric sequence**. J Mol Evol.

[39] Echeverria PC, Rojas PA, Martin V, Guarnera EA, Pszenny V, Angel SO (2000). **Characterisation of a novel interspersed*****Toxoplasma gondii*****DNA repeat with potential uses for PCR diagnosis and PCR-RFLP analysis**. FEMS Microbiol Lett.

[40] Braun L, Cannella D, Ortet P, Barakat M, Sautel CF, Kieffer S, Garin J, Bastien O, Voinnet O, Hakimi M-A (2010). **A complex small RNA repertoire is generated by a plant/fungal-like machinery and effected by a metazoan-like Argonaute in the single-cell human parasite*****Toxoplasma gondii***. PLoS Pathog.

[41] Howe DK, Sibley LD (1995). ***Toxoplasma gondii*****comprises three clonal lineages: correlation of parasite genotype with human disease**. J Infect Dis.

[42] Holm L (1986). **Codon usage and gene expression**. Nucleic Acids Res.

[43] McInerney JO (1998). **GCUA: general codon usage analysis**. Bioinformatics.

[44] Sharp PM, Bailes E, Grocock RJ, Peden JF, Sockett RE (2005). **Variation in the strength of selected codon usage bias among bacteria**. Nucleic Acids Res.

[45] Suzuki H, Brown CJ, Forney LJ, Top EM (2008). **Comparison of correspondence analysis methods for synonymous codon usage in bacteria**. DNA Res.

[46] Arnoult N, Van Beneden A, Decottignies A (2012). **Telomere length regulates TERRA levels through increased trimethylation of telomeric H3K9 and HP1alpha**. Nat Struct Mol Biol.

[47] Bah A, Azzalin CM (2012). **The telomeric transcriptome: from fission yeast to mammals**. Int J Biochem Cell Biol.

[48] Mancio-Silva L, Rojas-Meza AP, Vargas M, Scherf A, Hernandez-Rivas R (2008). **Differential association of Orc1 and Sir2 proteins to telomeric domains in*****Plasmodium falciparum***. J Cell Sci.

[49] Flueck C, Bartfai R, Niederwieser I, Witmer K, Alako BTF, Moes S, Bozdech Z, Jenoe P, Stunnenberg HG, Voss TS (2010). **A major role for the*****Plasmodium falciparum*****ApiAP2 protein PfSIP2 in chromosome end biology**. PLoS Pathog.

[50] Luke B, Lingner J (2009). **TERRA: telomeric repeat-containing RNA**. EMBO J.

[51] Peixoto L, Chen F, Harb OS, Davis PH, Beiting DP, Brownback CS, Ouloguem D, Roos DS (2010). **Integrative genomic approaches highlight a family of parasite-specific kinases that regulate host responses**. Cell Host Microbe.

[52] Pollard MA, Onatolu KN, Hiller L, Haldar K, Knoll LJ (2008). **Highly polymorphic family of glycosylphosphatidylinositol-anchored surface antigens with evidence of developmental regulation in*****Toxoplasma gondii***. Infect Immun.

[53] Calvin Jung, Cleo Y-F Lee, Grigg E Michael (2004). **The SRS superfamily of Toxoplasma surface proteins**. Int J Parasitol.

[54] Wasmuth JD, Pszenny V, Haile S, Jansen EM, Gast AT, Sher A, Boyle JP, Boulanger MJ, Parkinson J, Grigg ME: **Integrated bioinformatic and targeted deletion analyses of the SRS gene superfamily identify SRS29C as a negative regulator of*****Toxoplasma*****virulence****.** *MBio* 2012.,**3**(6)**:** doi:10.1128/mBio.00321–12 10.1128/mBio.00321-12 PMC3509429 23149485

[55] Hassan MA, Melo MB, Haas B, Jensen KDC, Saeij JPJ (2012). **De novo reconstruction of the*****Toxoplasma gondii*****transcriptome improves on the current genome annotation and reveals alternatively spliced transcripts and putative long non-coding RNAs**. BMC Genomics.

[56] Ferdig MT, Su XZ (2000). **Microsatellite markers and genetic mapping in*****Plasmodium falciparum***. Parasitol Today.

[57] Rudner R, Karkas JD, Chargaff E (1968). **Separation of B. subtilis DNA into complementary strands. 3 Direct analysis**. Proc Natl Acad Sci USA.

[58] Albrecht-Buehler G (2006). **Asymptotically increasing compliance of genomes with Chargaff’s second parity rules through inversions and inverted transpositions**. Proc Natl Acad Sci USA.

[59] Camacho C, Madden T, Ma N, Agarwala R, Morgulis A: **BLAST Command Line Applications User Manual. National Center for Biotechnology Information (US), (2008)****.** National Center for Biotechnology Information (US). Camacho C, Madden T, Coulouris G, et al. BLAST Command Line Applications User Manual. 2008 Jun 23 [Updated 2013 Mar 25]. In: BLAST Help [Internet]. Bethesda (MD): National Center for Biotechnology Information (US); 2008-. Available from: [http://www.ncbi.nlm.nih.gov/books/NBK1763/]

[60] James C Abbott, David M Aanensen, Stephen D Bentley (2007). **WebACT: an online genome comparison suite**. Methods Mol Biol.

[61] Carver TJ, Rutherford KM, Berriman M, Rajandream MA, Barrell BG, Parkhill J (2005). **ACT: the Artemis Comparison Tool**. Bioinformatics.

[62] Krzywinski M, Schein J, Birol I, Connors J, Gascoyne R, Horsman D, Jones SJ, Marra MA (2009). **Circos: an information aesthetic for comparative genomics**. Genome Res.

[63] Greenacre MJ (2007). **Correspondence Analysis in Practice, 2nd edn. Interdisciplinary Statistics**.

