# Supplementary figures and images for: Characterization of Toxoplasma gondii subtelomeric-like regions: identification of a long-range compositional bias that is also associated with gene-poor regions
Source: BMC Genomics. 2014 Jan 13;15(1):21. doi: 10.1186/1471-2164-15-21 (PMC4008256; doi:10.1186/1471-2164-15-21)

A

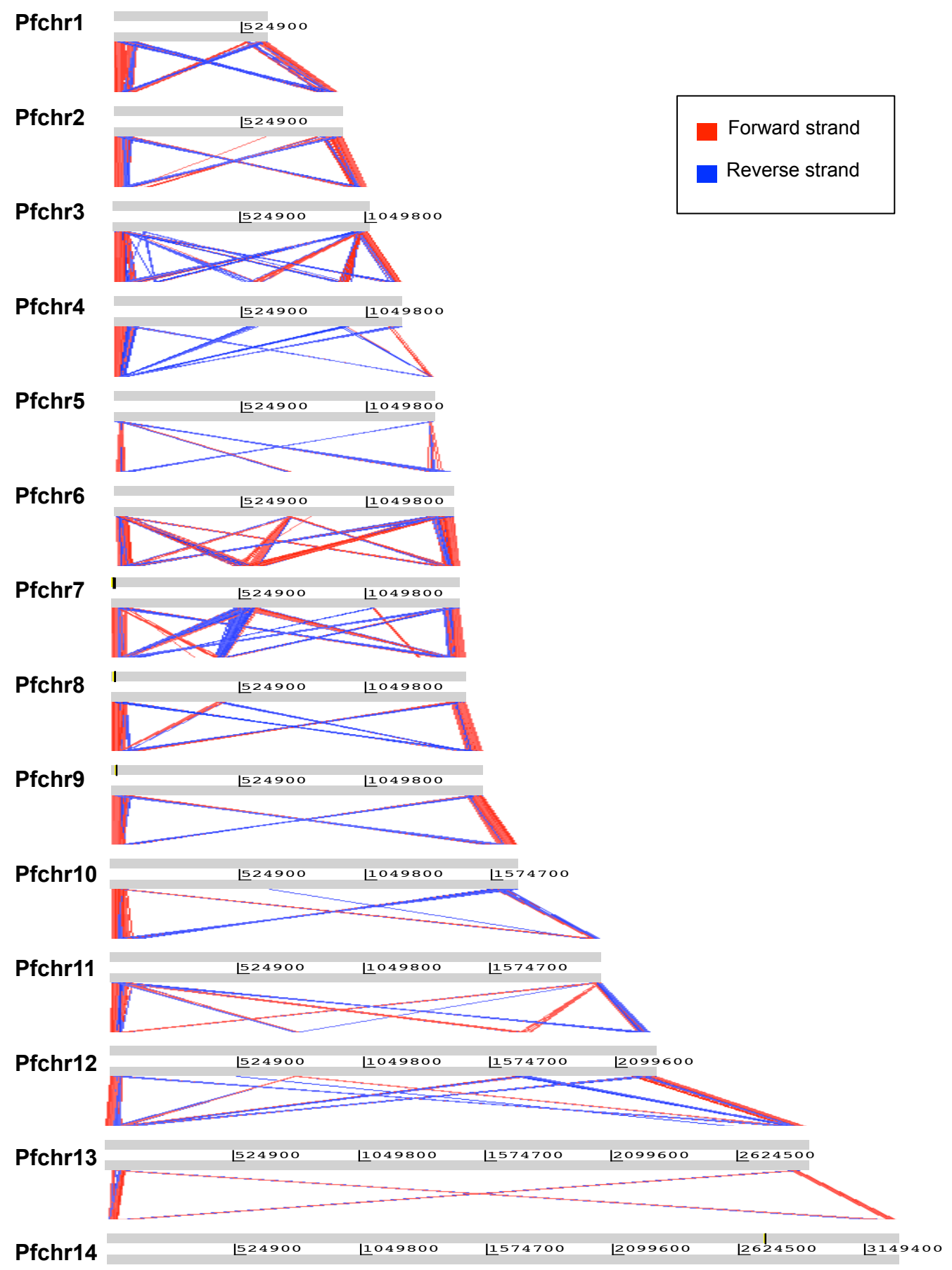

**B**

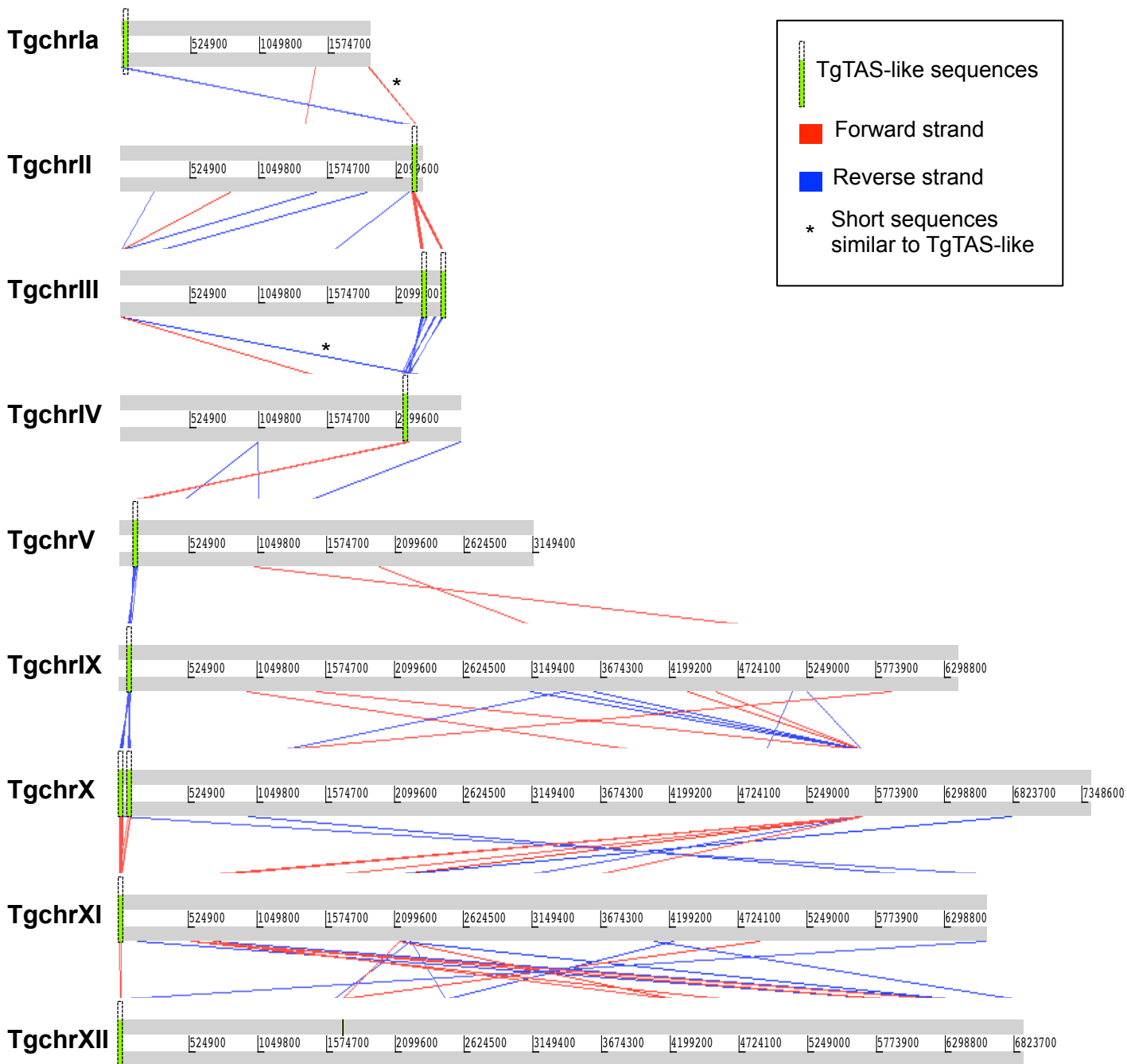

Supplement: Supplementary file 1 — 1: Pairwise comparisons between chromsomes. ACT visualization of chromosome similarities. The representation only shows the similarities between each chromosome (double grey lines, with coordinates in bp) and the contiguous chromosome, above or below [61]. Links in red genomic are genomic segments on forward strand, and in blue genomic segment on reverse strand. A. All P. falciparum chromosomes, not being filtered. B. T. gondii chromosomes containing TgTASL sequences (green box with a dashed line). The asterisks point to short TgTASL sequences present in other chromosomal regions. Sequences were filtered to be at least 180 bp long. (PDF 160 KB) [file 12864_2013_7001_MOESM1_ESM.pdf]

**A****TgTAS-like**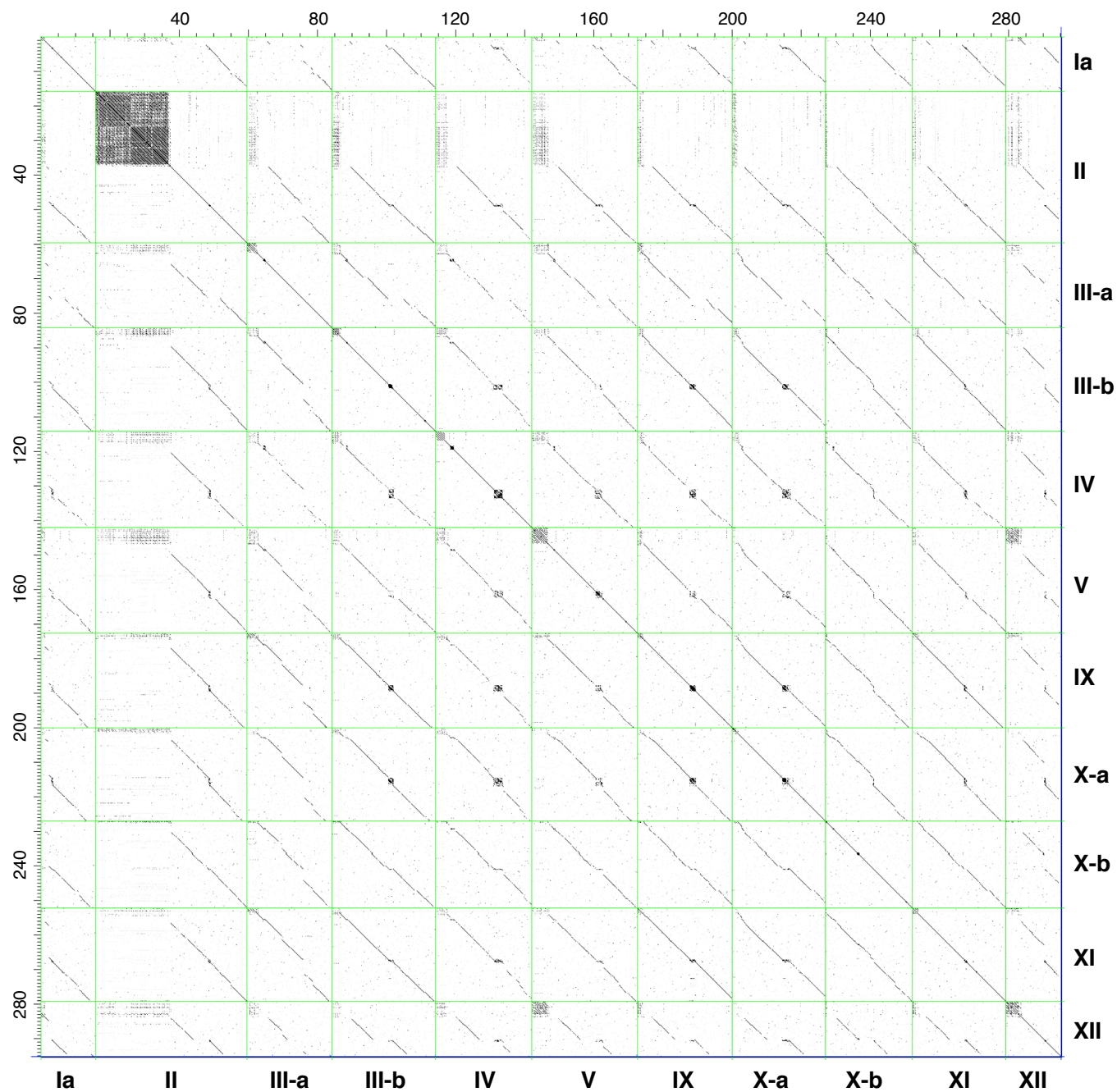**B**

Similarities between TgTASL\_IV and other TgTASL

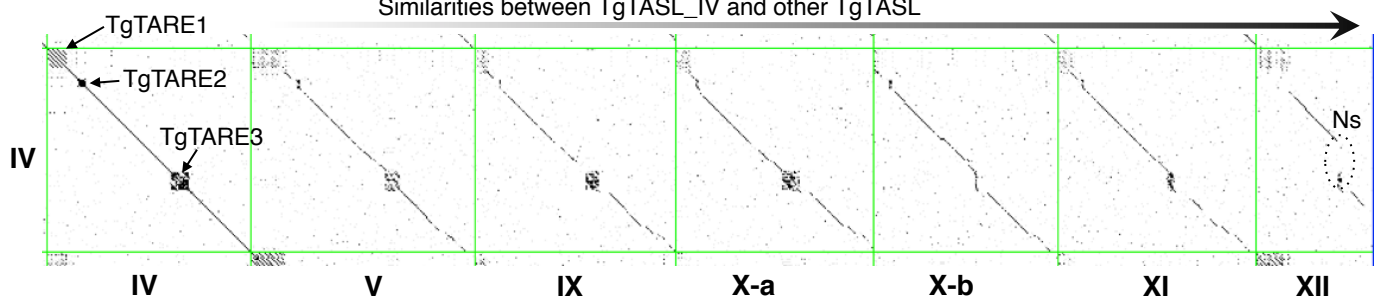

Supplement: Supplementary file 2 — Additional file 2: All-vs-all dotplot comparisons of TgTAS-like regions. Dotplots were generated from a multifasta file containing all TgTAS- like regions. The green lines separate each TgTASL. A. The complete dotplot of all TgTAS-like regions in the ME49 strain. The axes on the top and left show the size in Kb, whereas the ones on the bottom and the right shows the name of each TgTASL being compared. B. Schematic representation of the dotplot analysis, using part of a row of TgTASL_IV. In the first panel the TgTASL_IV region was compared with itself, clearly showing the three blocks of repeats (TgTARE 1 to 3) being represented by several lines (one per repeat). In the rest of the panels TgTASL_IV was compared with TgTASL_V to _XII showing similarities among these TgTASL regions. Missing sequences, insertions and deletions are visible as gaps in the sequence comparison, for example in the last panel, where a run of Ns connecting two contigs in TgTASL_XII appears as a large gap in the dotplot. (PDF 1 MB) [file 12864_2013_7001_MOESM2_ESM.pdf]

**chrIa**

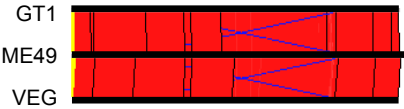

**chrII**

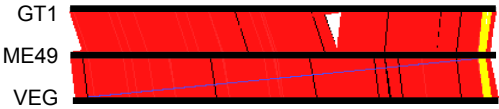

**chrIII**

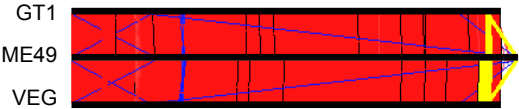

**chrIV**

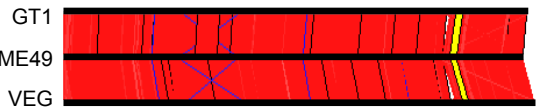

**chrV**

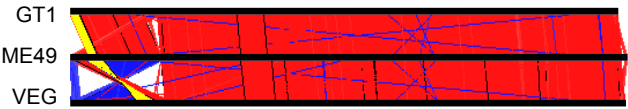

**chrVI**

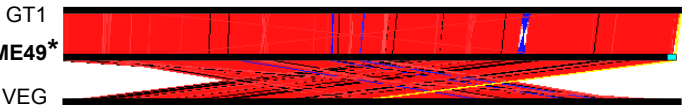

**chrIX**

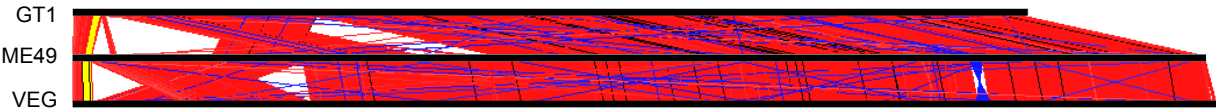

**chrX**

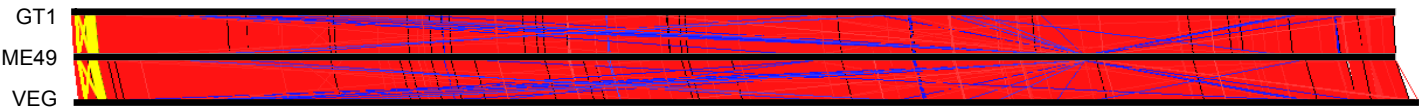

**chrXI**

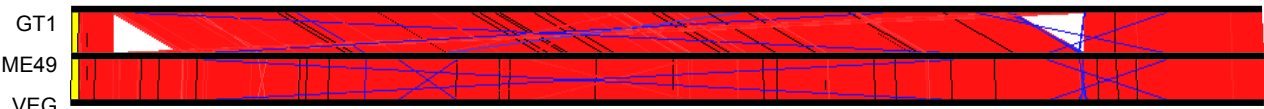

**chrXII**

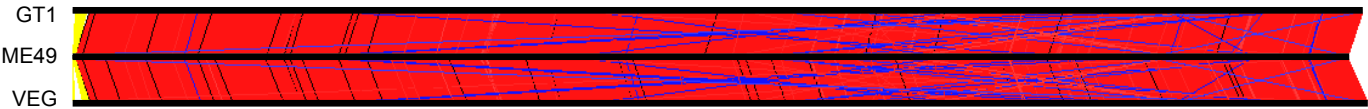

500 Kb

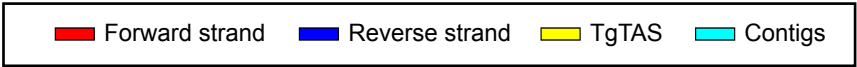

Supplement: Supplementary file 4 — Additional file 4: Comparative genomics of different T. gondii strains. Chromosomal similarity visualizations using ACT. BLASTN similarities across chromosomes are shown as red (forward strand), or blue (reverse) segments. Similarity between TgTAS regions is shown with yellow segments. The figure shows a comparative genomics analysis of all chromosomes containing TgTASL in three T. gondii strains: GT1 (type I), ME49 (type II) and VEG (type III). The asterisk next to the ME49 chromosome VI is indicating that at the end of the chromosome there are three additional contigs (light blue) containing sequences similar to TgTASL_VI in the GT1 and VEG strains. (PDF 544 KB) [file 12864_2013_7001_MOESM4_ESM.pdf]

# NcTASL vs TgTASL

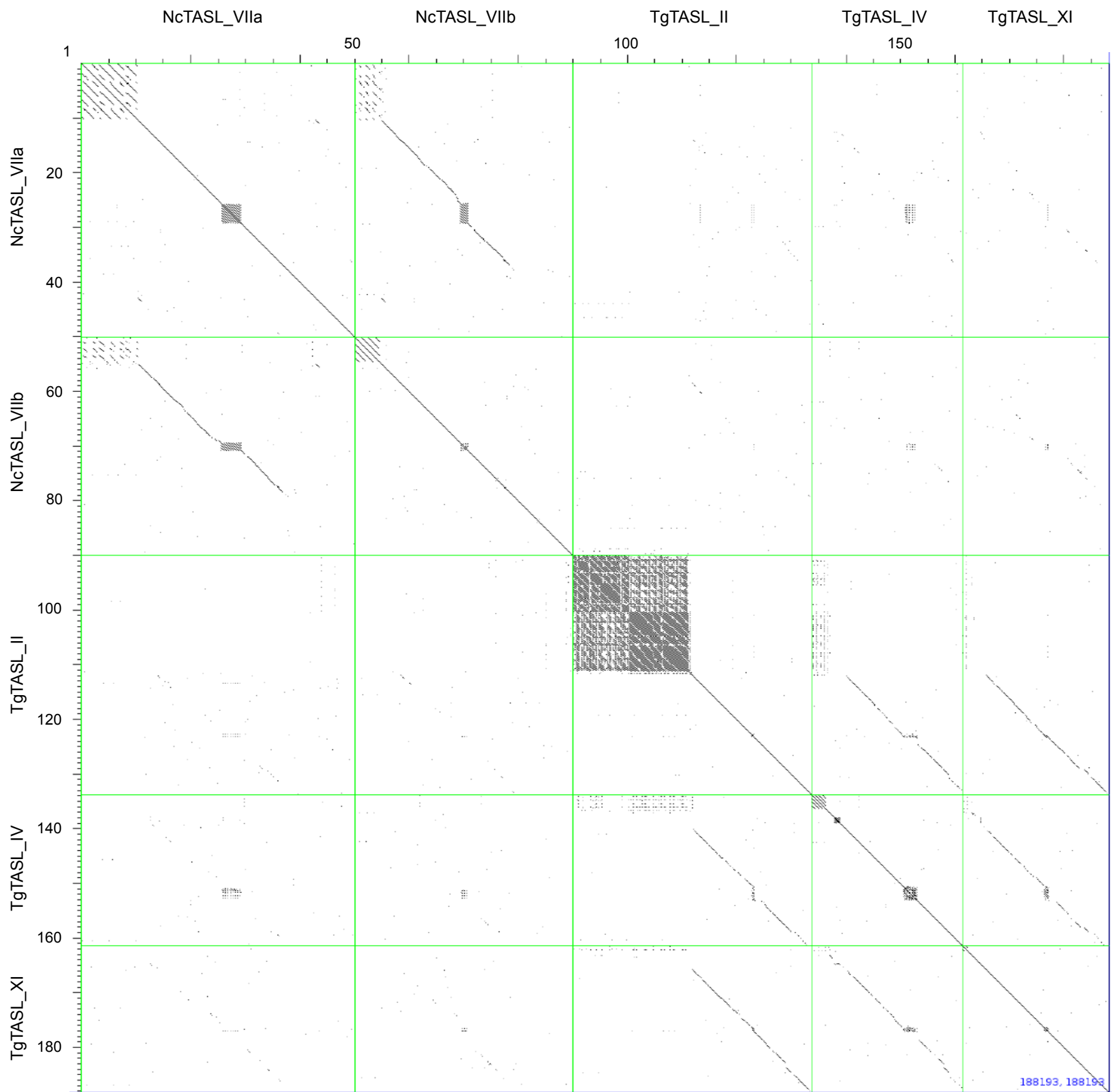

Supplement: Supplementary file 5 — Additional file 5: Dotplot of Neospora caninum vs T. gondii TAS-like sequences. The presence of conserved patterns in the NcTASL, and their lack of similarity against TgTASL, were evaluated by all-vs-all pairwise comparison using Dotter. The dotplot includes the 2 putative NcTASL and 3 representative TgTASL. (PDF 159 KB) [file 12864_2013_7001_MOESM5_ESM.pdf]

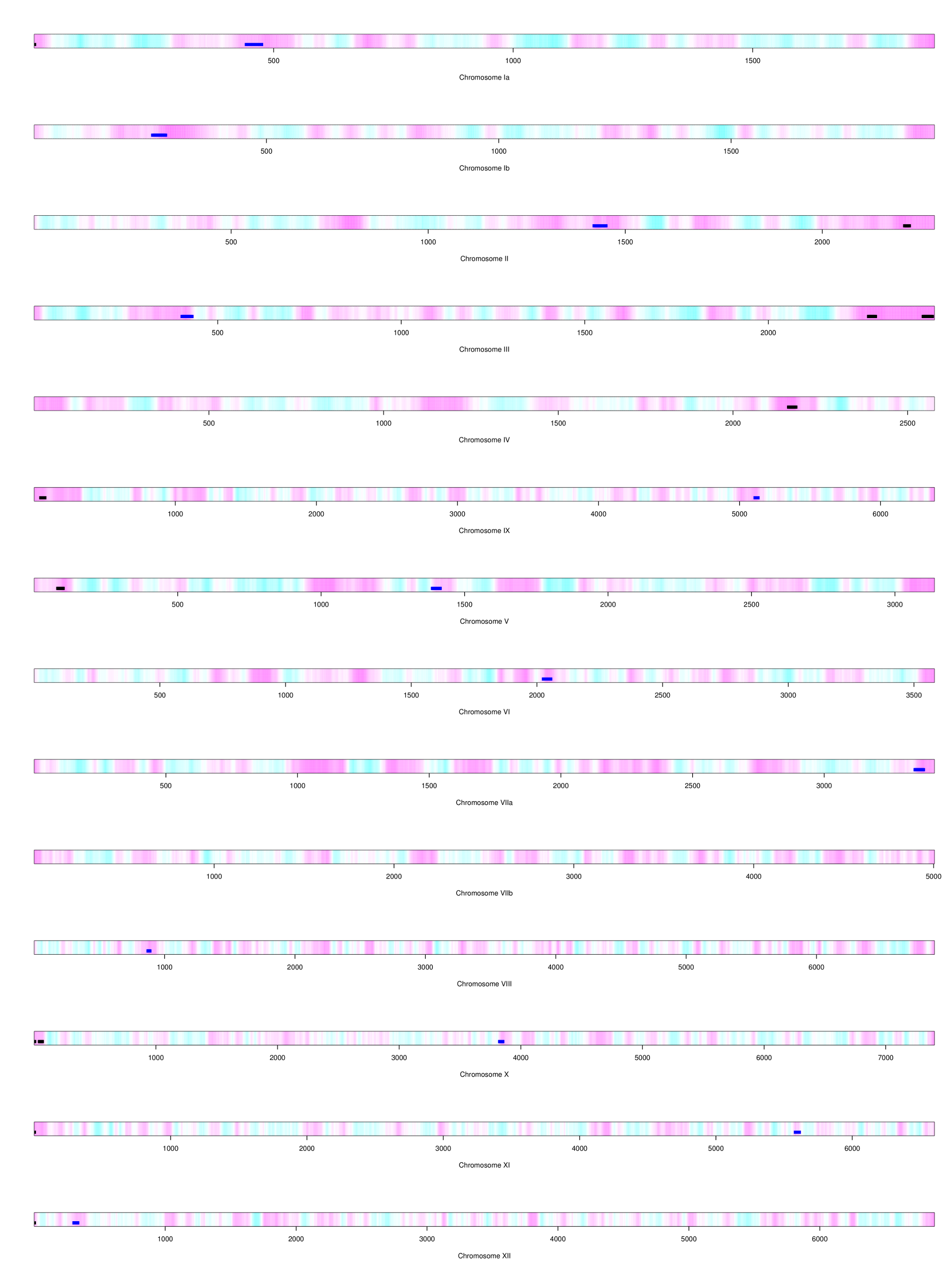

Supplement: Supplementary file 7 — Additional file 7: Visualization of TgTAS-like compositional bias along Toxoplasma chromosomes. A schematic representation of chromosomes is depicted where the major trend in trinucleotide compositional bias (first principal coordinate at N = 40 Kb) is encoded with a color gradient going from cyan (negative values in 1st coordinate, see Figure 4) to magenta (positive values in 1st coordinate), and passing through white (zero, no bias). The position of TgTAS-like and centromeric regions are marked with black and blue boxes, respectively. (TIFF 1 MB) [file 12864_2013_7001_MOESM7_ESM.tiff]

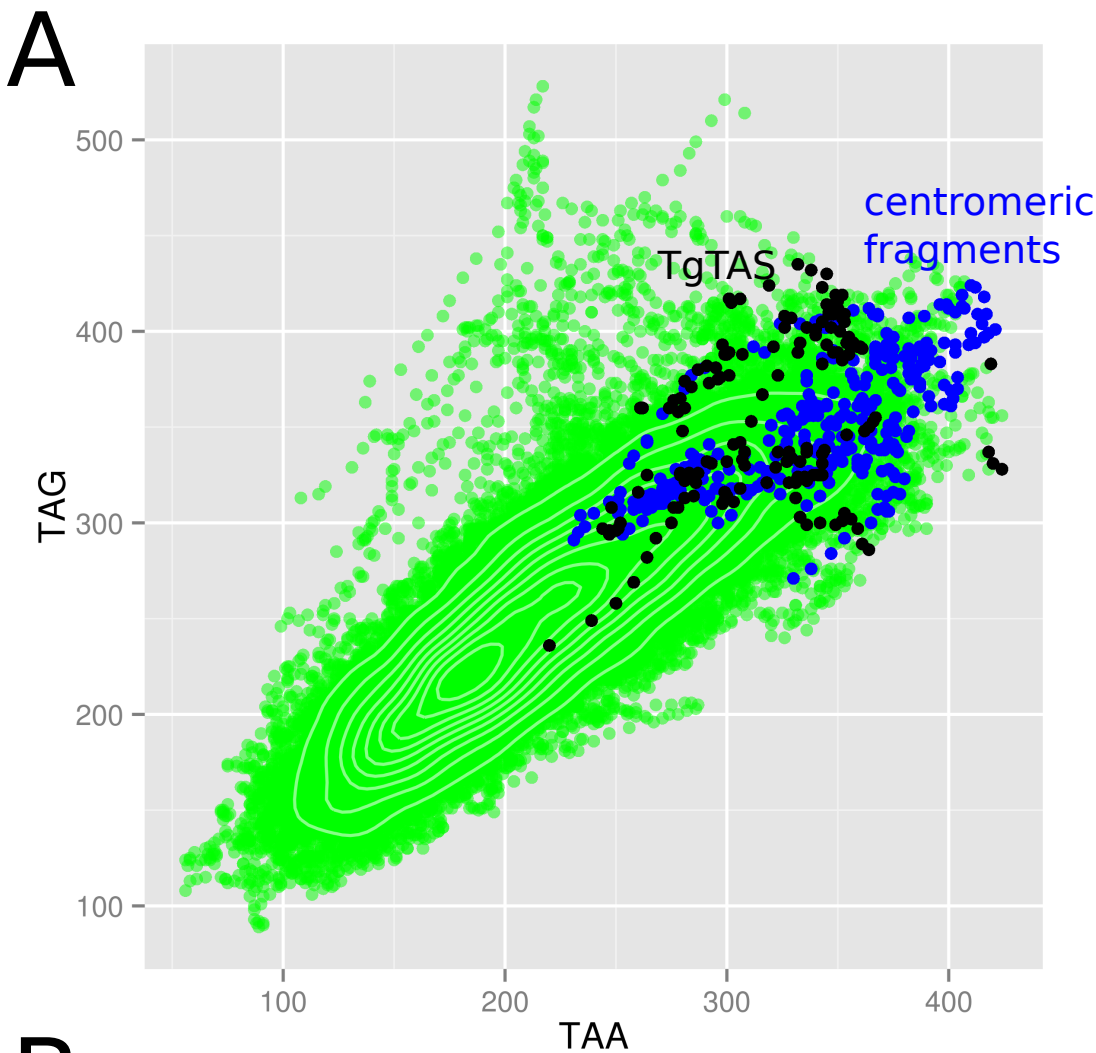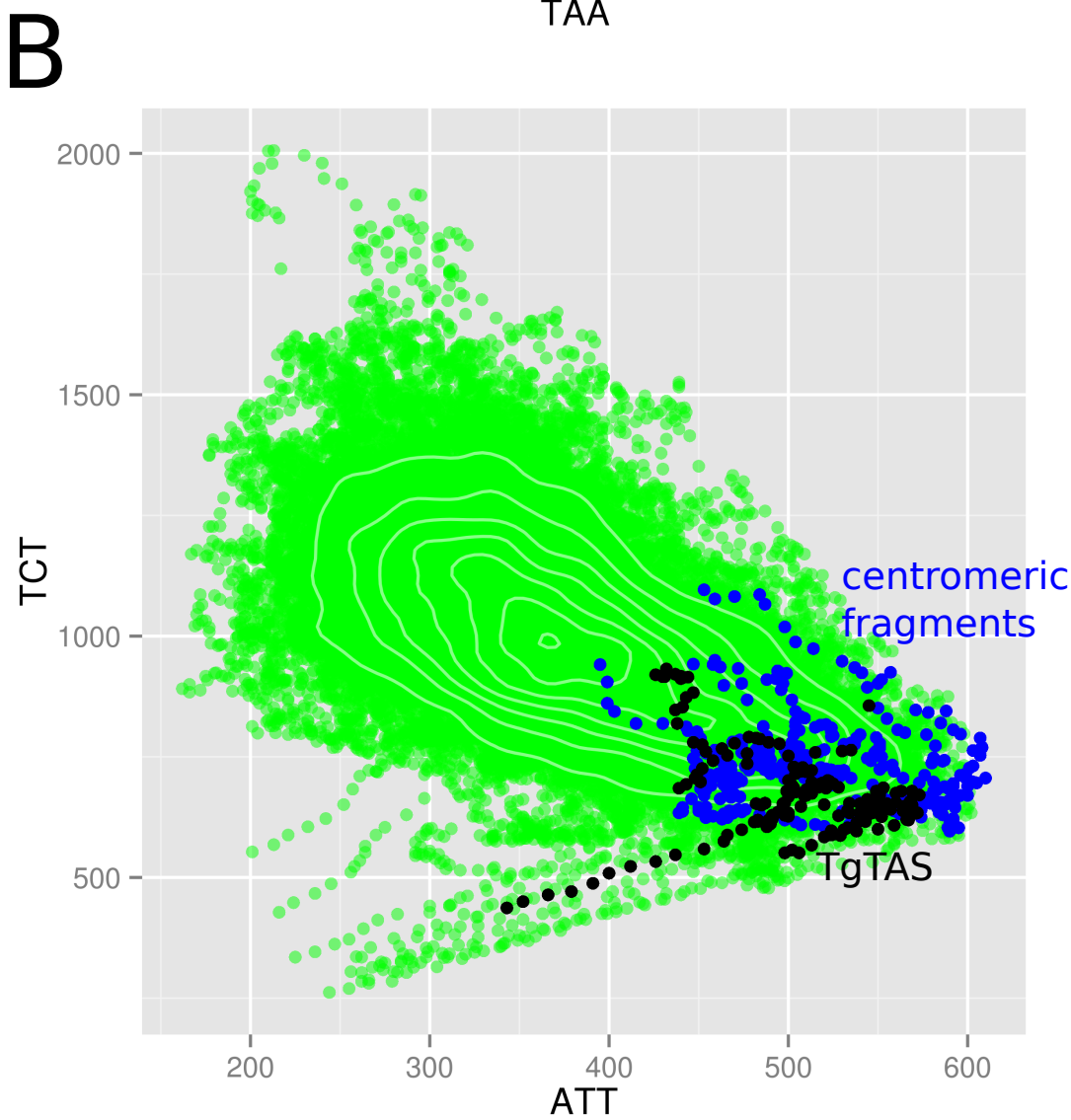

Supplement: Supplementary file 8 — Additional file 8: Example scatterplots of trinucleotide abundance. These plots show the distribution of all 40 Kb genomic fragments according to trinucleotide counts of selected trinucleotides. A. This panel shows a symetric biplot for two pairs of trinucleotides: TAA/TTA and TAG/CTA, which are read as STOP codons in coding sequences. These contribute with a 12.7% of the major bias trend when considering all trinucleotides (see text). B. This panel shows a symetric biplot for two other influent trinucleotides ATT/AAT and TCT/AGA, contributing with a 19% of the major trend. The two axes show the number of trinucleotide counts in a window of 40 Kb. In both plots the fragments containing TgTAS-like regions are displayed in black and those containing centromeric fragments in blue. (PDF 829 KB) [file 12864_2013_7001_MOESM8_ESM.pdf]
