# Supplementary material for: Characterization of Toxoplasma gondii subtelomeric-like regions: identification of a long-range compositional bias that is also associated with gene-poor regions
Source: BMC Genomics. 2014 Jan 13;15(1):21. doi: 10.1186/1471-2164-15-21 (PMC4008256; doi:10.1186/1471-2164-15-21)
Supplement: Supplementary file 6 — Additional file 6: Correspondence analysis Maps of genomic fragments of 1 to 80 Kb. This supplementary file contains symmetric biplots similar to those in Figure 4. The PDF file contains a succession of maps obtained for increasing genomic window sizes. (PDF 5 MB) [file 12864_2013_7001_MOESM6_ESM.pdf]

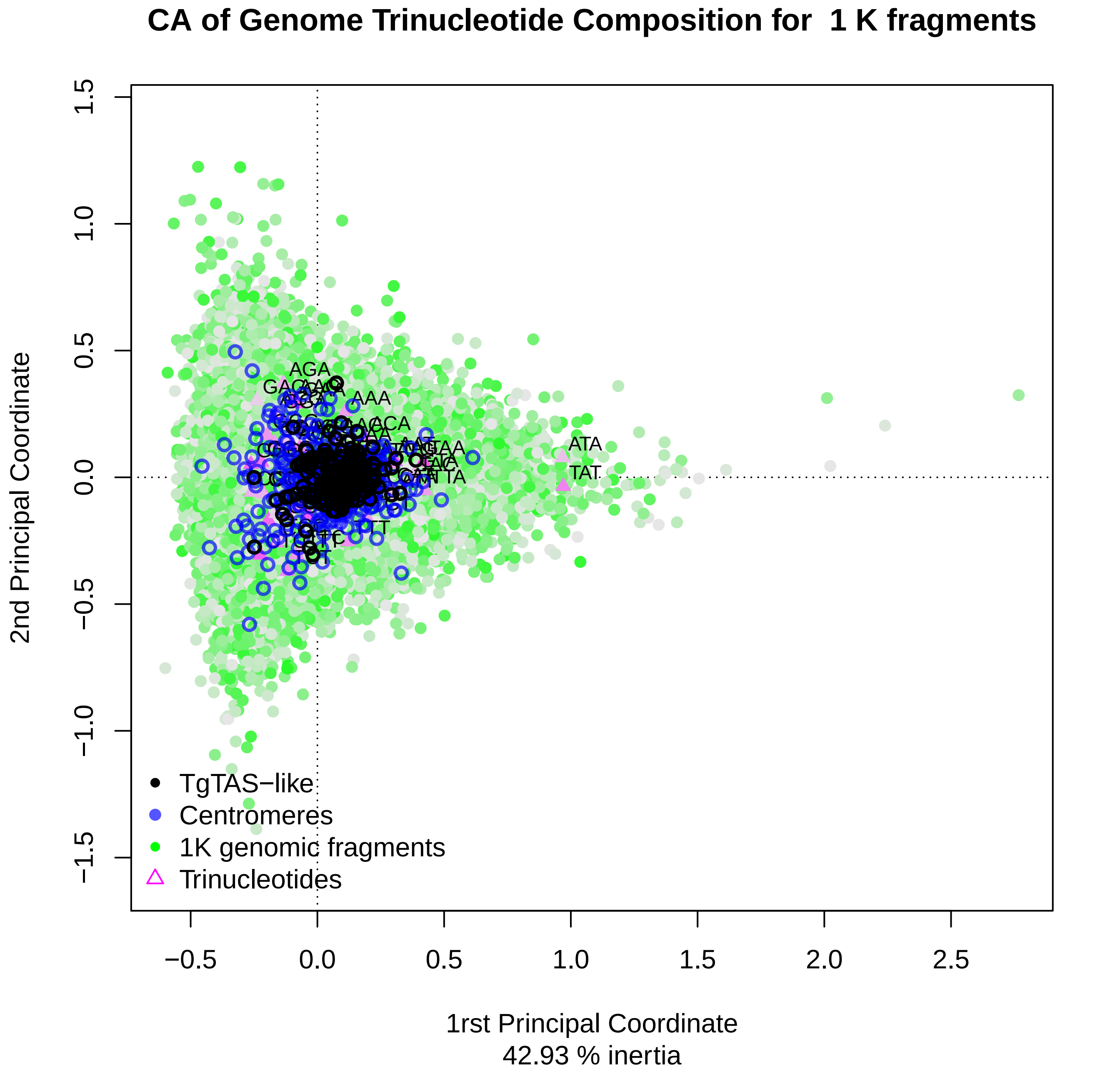
CA of Genome Trinucleotide Composition for 1 K fragments
2nd Principal Coordinate
1rst Principal Coordinate
42.93 % inertia
1.5
1.0
0.5
0.0
−0.5
−1.0
−1.5
2.0
2.5
AGA
AAA
ACA
ATA
TAT
TgTAS−like
Centromeres
1K genomic fragments
Trinucleotides

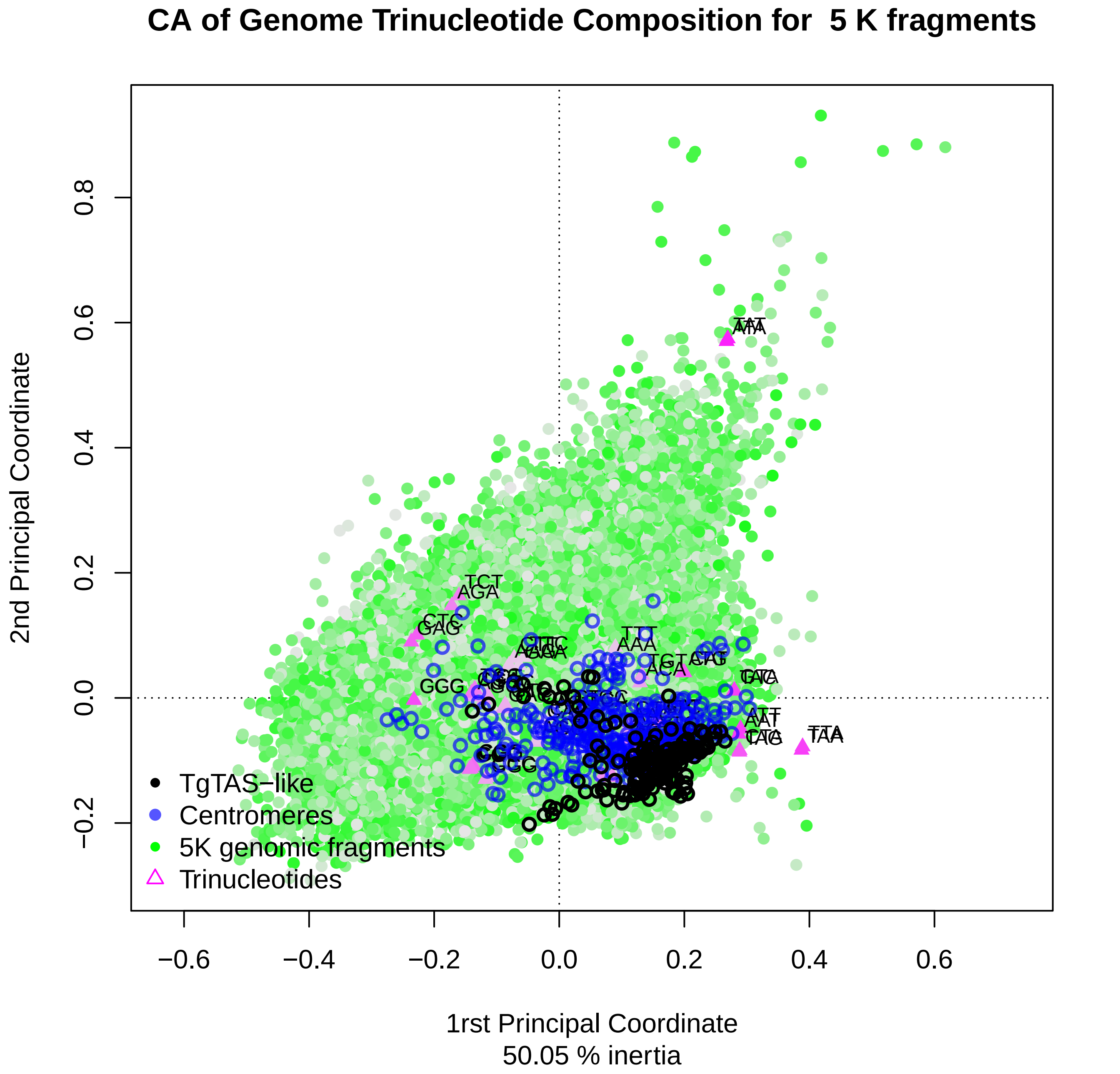
CA of Genome Trinucleotide Composition for 5 K fragments
2nd Principal Coordinate
1rst Principal Coordinate
50.05 % inertia
−0.6
−0.4
−0.2
0.0
0.2
0.4
0.6
0.8
TgTAS−like
Centromeres
5K genomic fragments
Trinucleotides
TCT
AGA
GAG
GGG
TTT
AAA
TAA

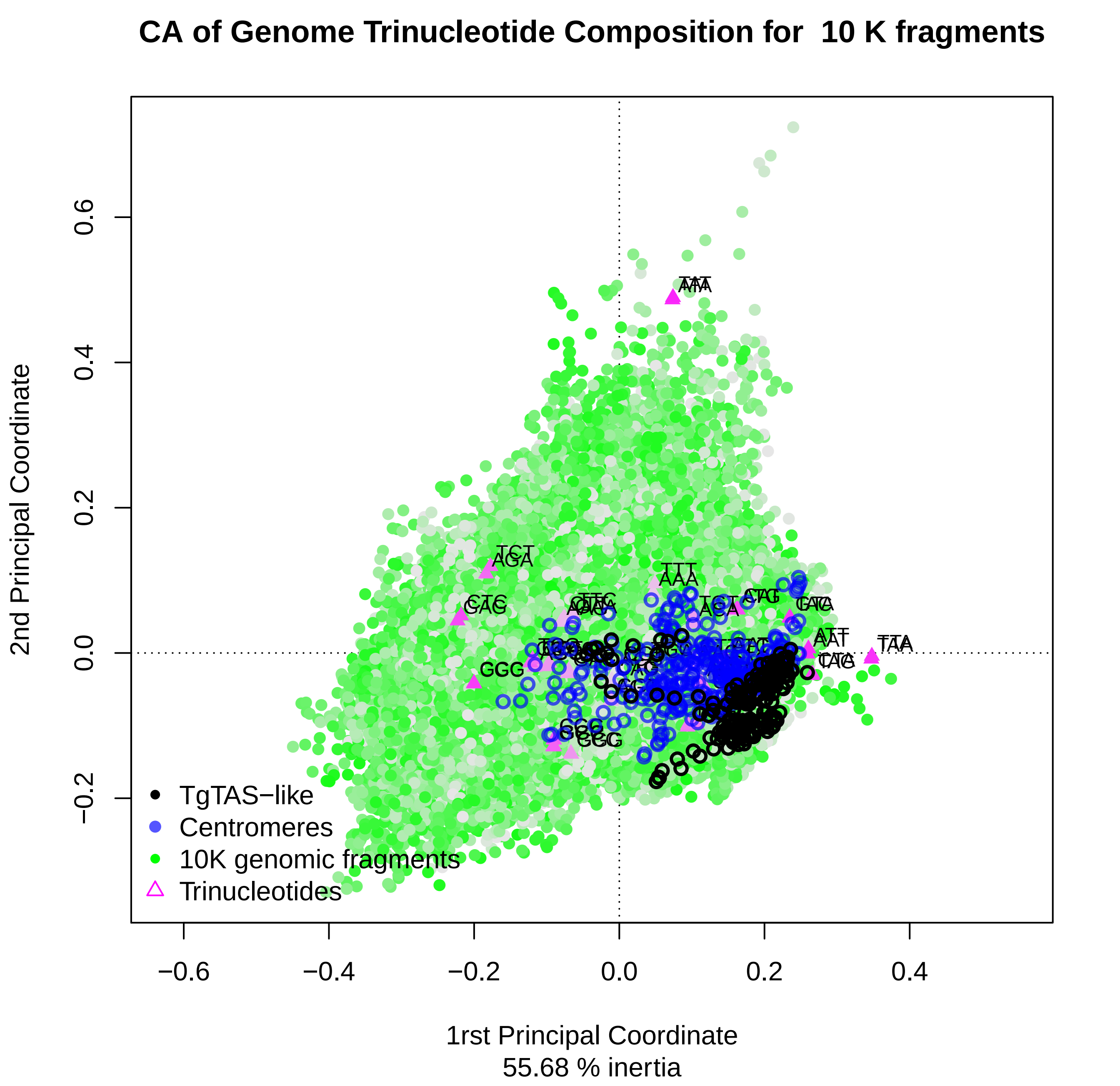
CA of Genome Trinucleotide Composition for 10 K fragments
2nd Principal Coordinate
0.6
0.4
0.2
0.0
−0.2
−0.6
−0.4
−0.2
0.0
0.2
0.4
1rst Principal Coordinate
55.68 % inertia
TgTAS−like
Centromeres
10K genomic fragments
Trinucleotides

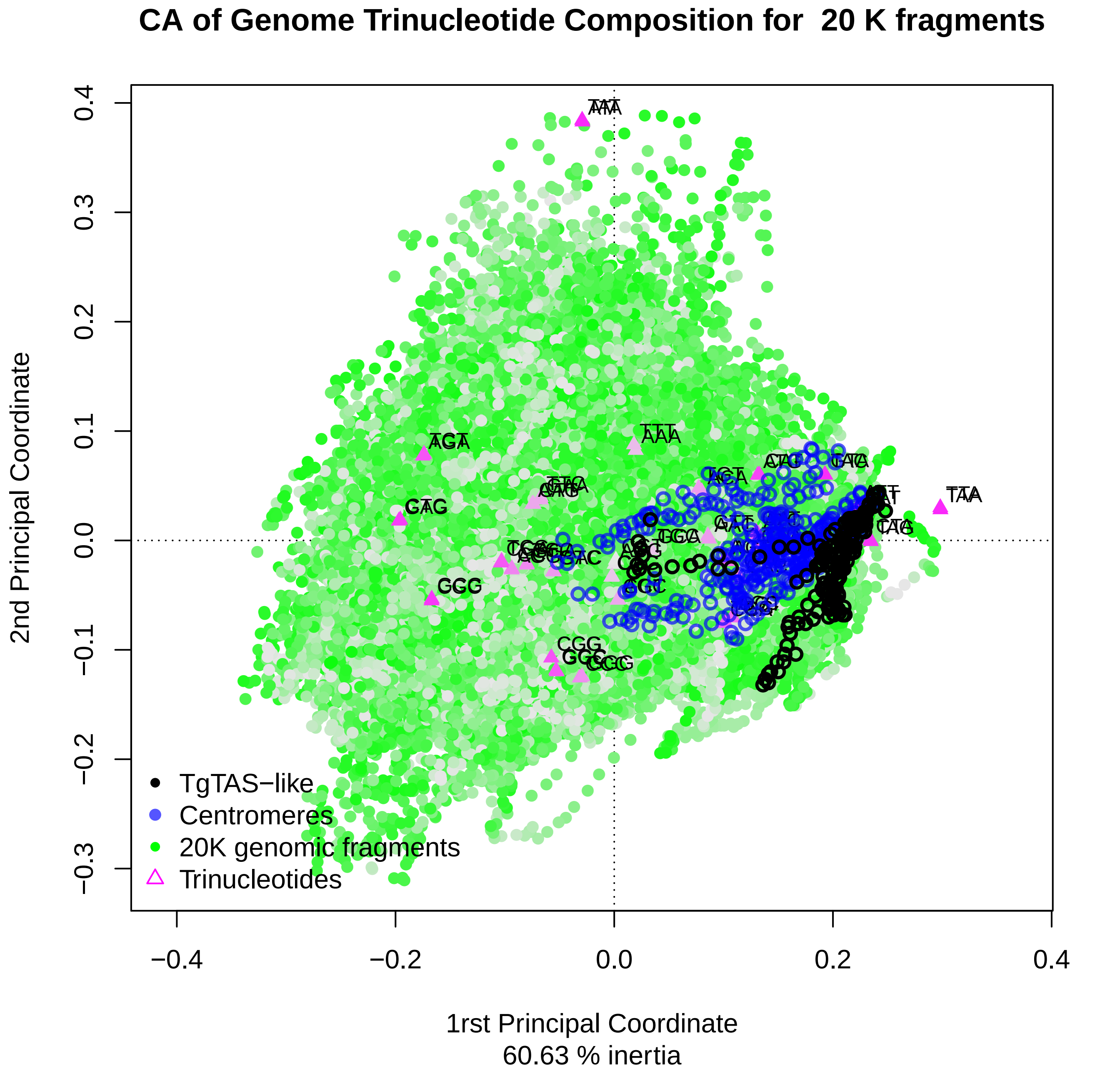
CA of Genome Trinucleotide Composition for 20 K fragments
2nd Principal Coordinate
1rst Principal Coordinate
60.63 % inertia
0.4
0.3
0.2
0.1
0.0
−0.1
−0.2
−0.3
−0.4
TgTAS−like
Centromeres
20K genomic fragments
Trinucleotides
GGG
TAA
CGG

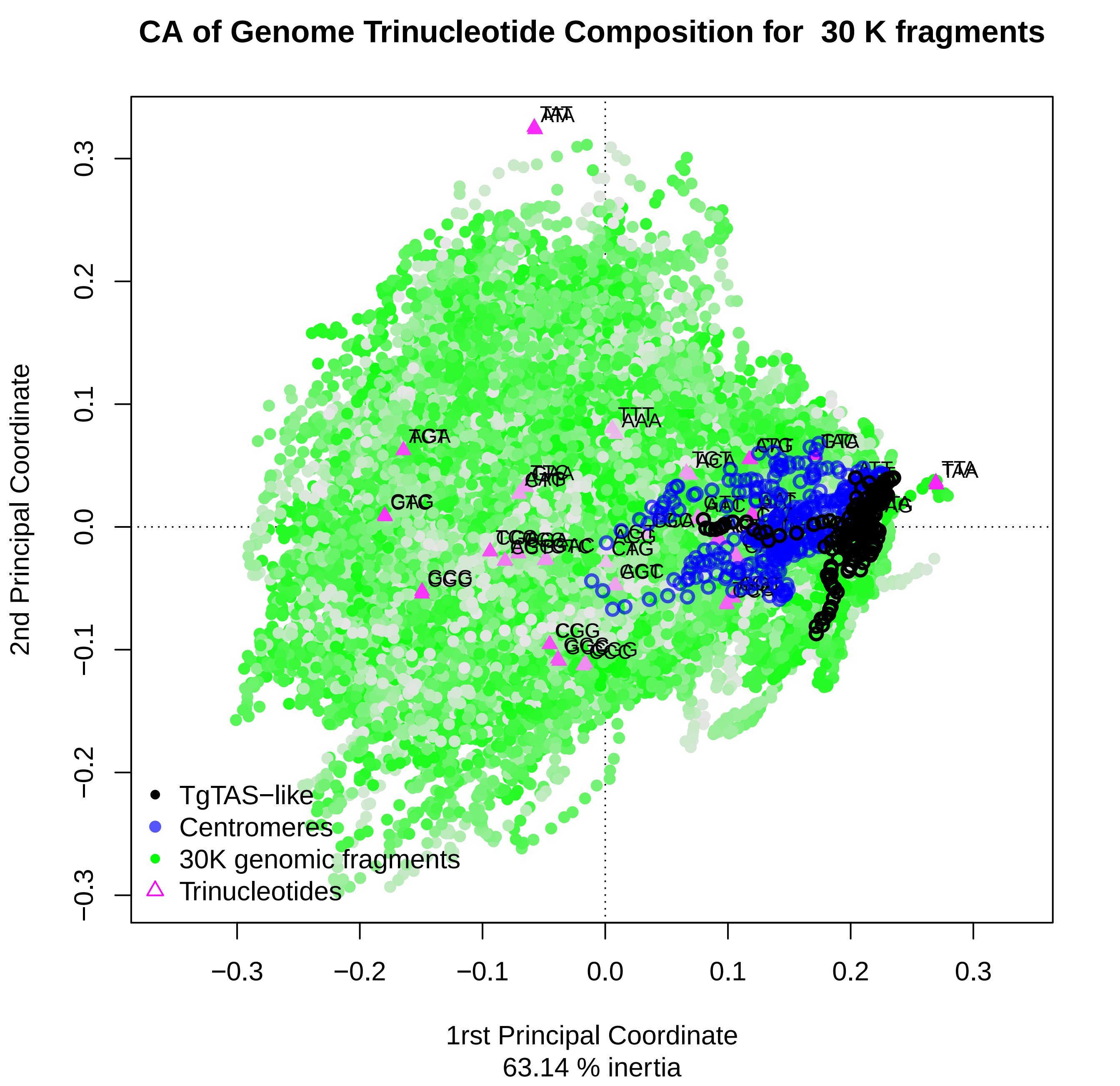

CA of Genome Trinucleotide Composition for 30 K fragments
2nd Principal Coordinate
1rst Principal Coordinate
63.14 % inertia
0.3
0.2
0.1
0.0
−0.1
−0.2
−0.3
TgTAS−like
Centromeres
30K genomic fragments
Trinucleotides
TTA
TAA
GGG
CGG
CCG
AGT
CAG

CA of Genome Trinucleotide Composition for 40 K fragments

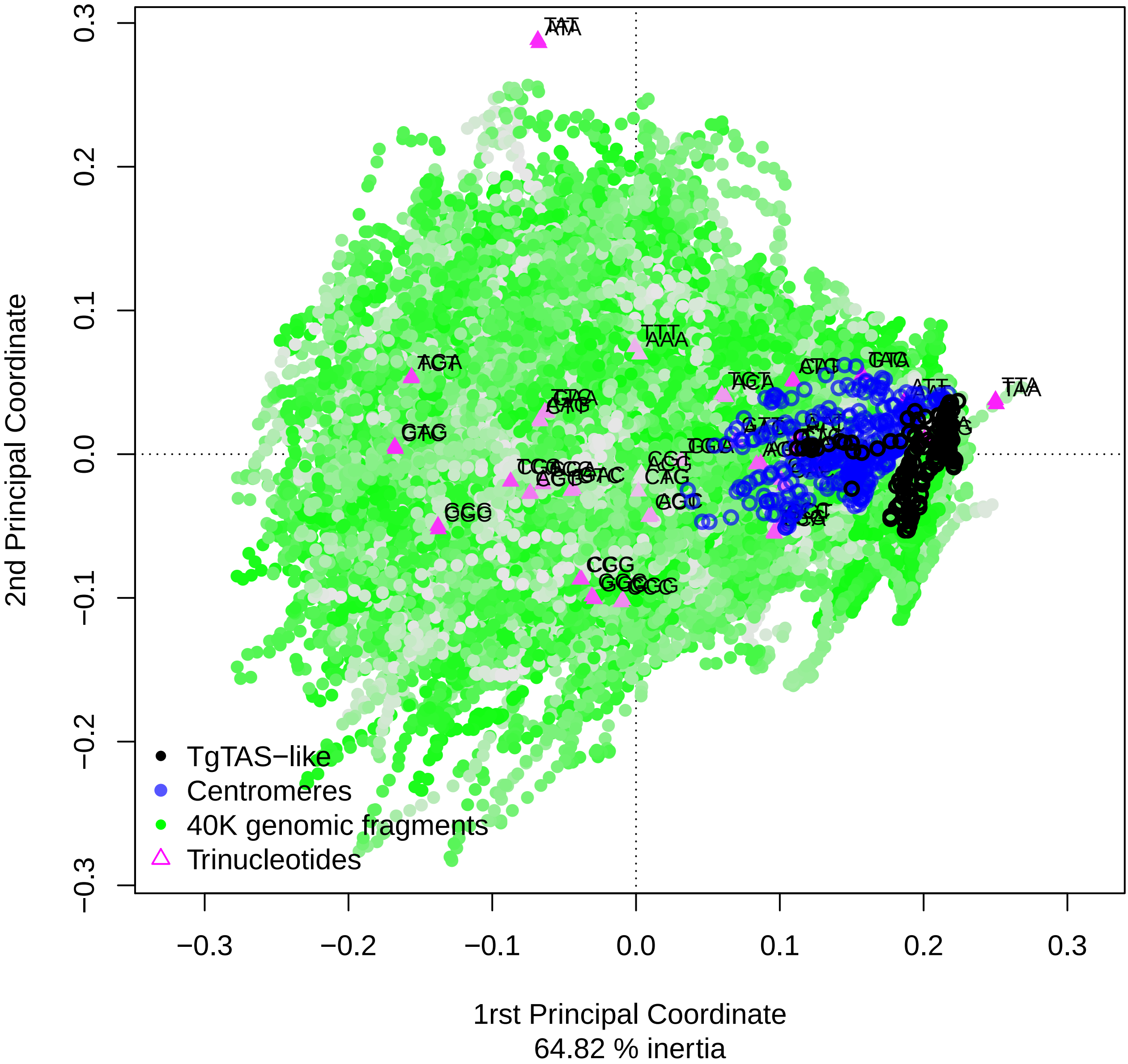

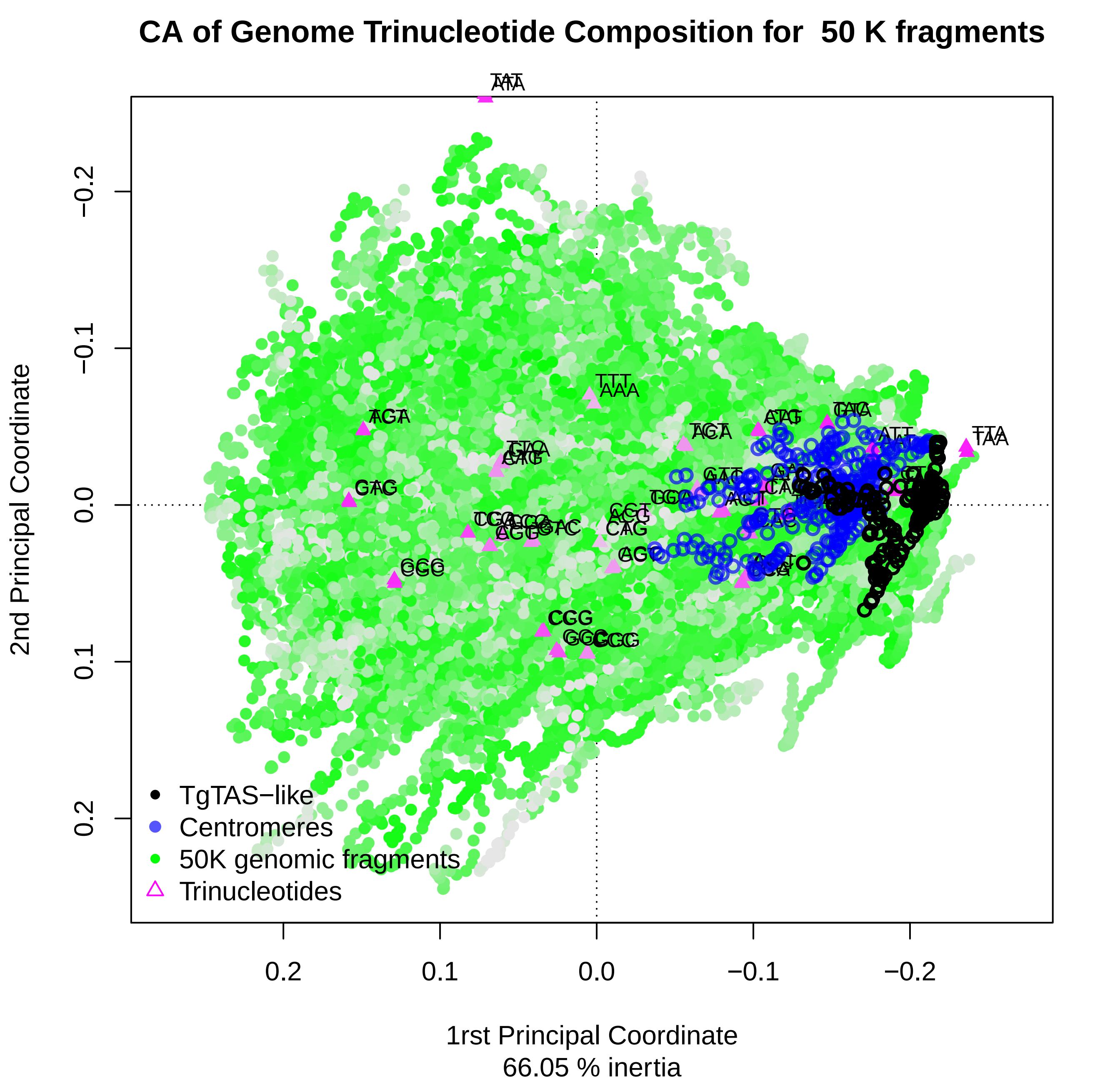
CA of Genome Trinucleotide Composition for 50 K fragments
2nd Principal Coordinate
−0.2
−0.1
0.0
0.1
0.2
1rst Principal Coordinate
66.05 % inertia
TgTAS−like
Centromeres
50K genomic fragments
Trinucleotides

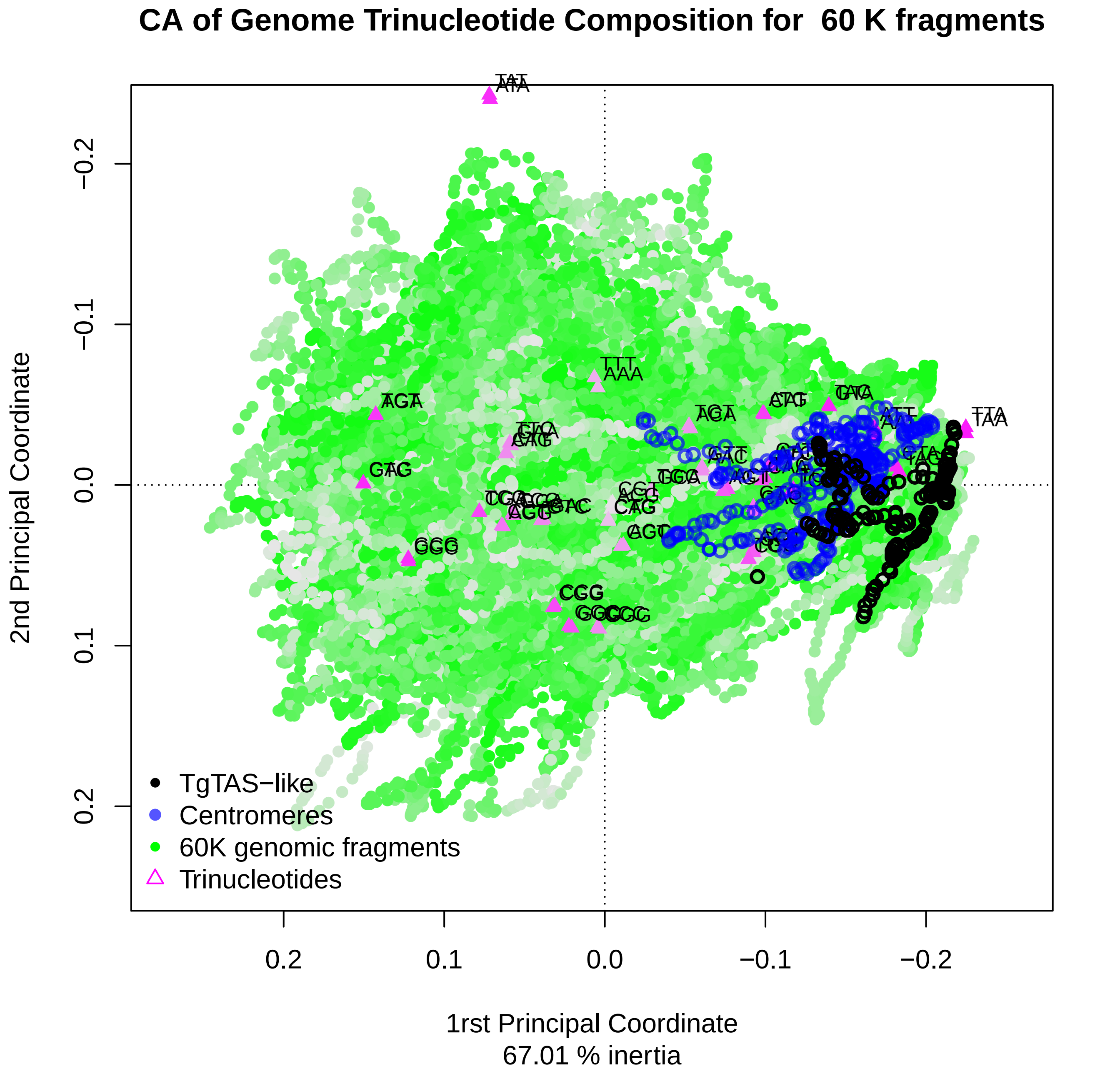

CA of Genome Trinucleotide Composition for 60 K fragments
2nd Principal Coordinate
1rst Principal Coordinate
67.01 % inertia
−0.2
−0.1
0.0
0.1
0.2
TgTAS−like
Centromeres
60K genomic fragments
Trinucleotides

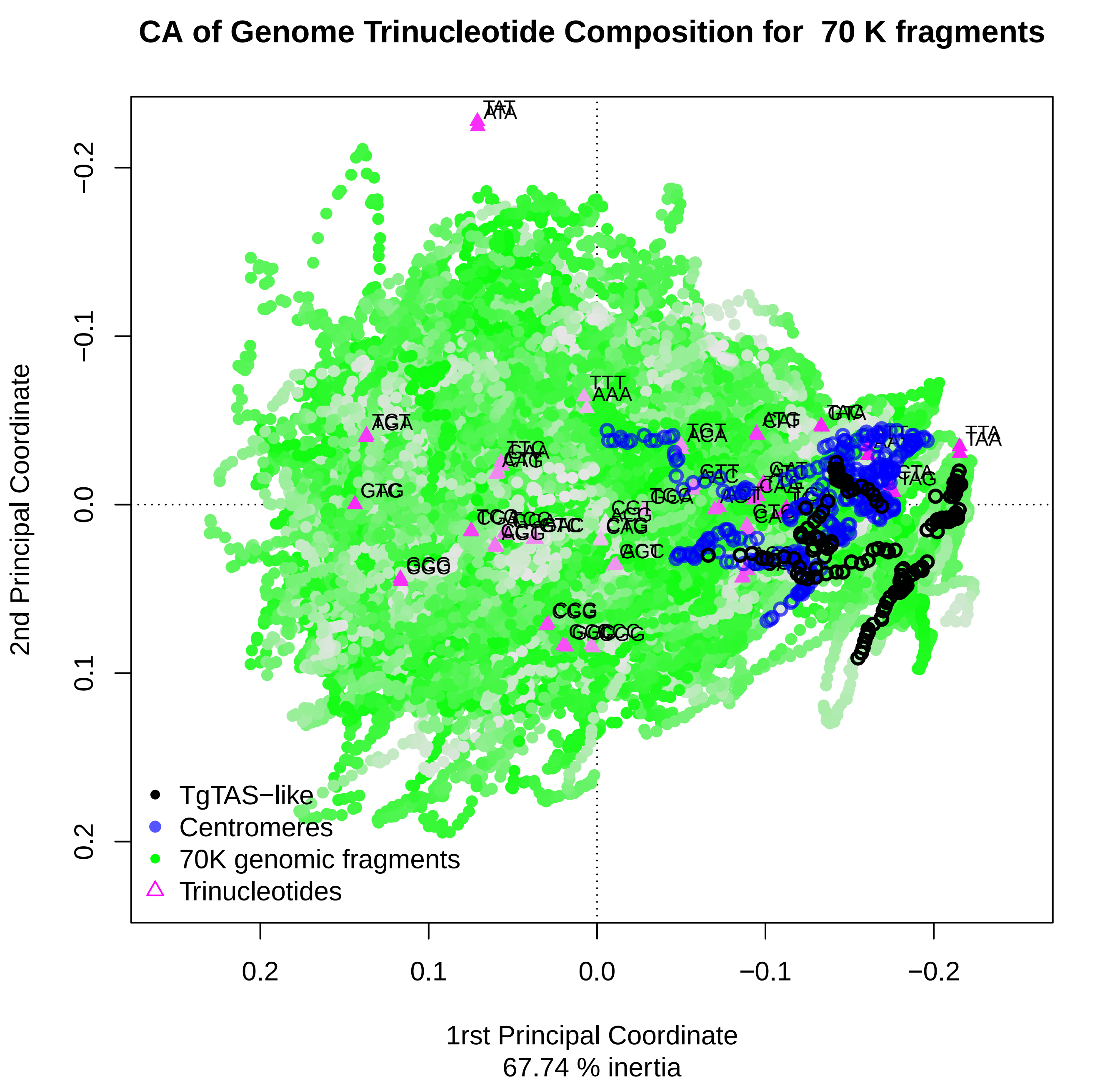
CA of Genome Trinucleotide Composition for 70 K fragments
2nd Principal Coordinate
1rst Principal Coordinate
67.74 % inertia
TgTAS−like
Centromeres
70K genomic fragments
Trinucleotides
−0.2
−0.1
0.0
0.1
0.2
0.2
0.1
0.0
−0.1
−0.2

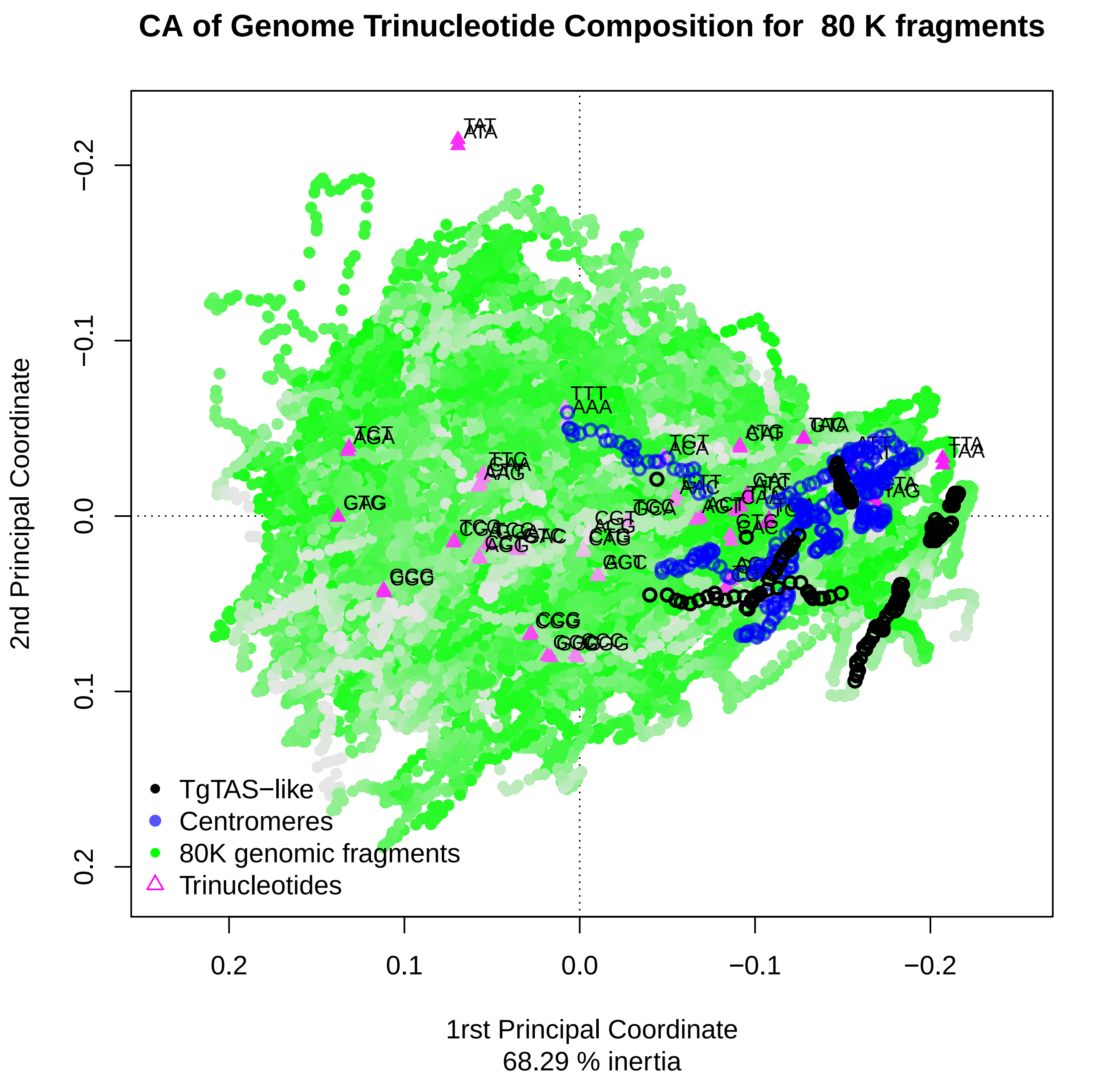
CA of Genome Trinucleotide Composition for 80 K fragments
2nd Principal Coordinate
−0.2
−0.1
0.0
0.1
0.2
1rst Principal Coordinate
68.29 % inertia
TgTAS−like
Centromeres
80K genomic fragments
Trinucleotides
